# A Differential Hypofunctionality of Gαi Proteins Occurs in Adolescent Idiopathic Scoliosis and Correlates with the Risk of Disease Progression

**DOI:** 10.1038/s41598-019-46325-2

**Published:** 2019-07-11

**Authors:** Marie-Yvonne Akoume, Mohamed Elbakry, Maxime Veillette, Anita Franco, Dina Nada, Hubert Labelle, Jean-Marc Mac-Thiong, Guy Grimard, Jean Ouellet, Stefan Parent, Charles-Hilaire Rivard, Giovanni Lombardi, Alessandra Colombini, Giuseppe Banfi, Marco Brayda-Bruno, Kristen F. Gorman, Alain Moreau

**Affiliations:** 1 Viscogliosi Laboratory in Molecular Genetics of Musculoskeletal Diseases, Sainte-Justine University Hospital Research Center, Montréal, Quebec Canada; 2 Department of Pharmacology and Toxicology, Faculty of Medicine, Université des Sciences de la Santé (USS) de Libreville, Libreville, Gabon; 3 Biochemistry section, Chemistry Department, Faculty of Science, Tanta University, Tanta, Egypt; 4 Department of Biochemistry and Molecular Medicine, Faculty of Medicine, Université de Montréal, Montréal, Quebec Canada; 5 LIS3D Laboratory, Sainte-Justine University Hospital Research Center, Montréal, Quebec Canada; 6 Orthopedic Division, Sainte-Justine University Hospital and Department of Surgery, Faculty of Medicine, Université de Montréal, Montréal, Quebec Canada; 7 Orthopedic Division, The Montreal Children’s Hospital, Department of Surgery, McGill University, Montréal, Quebec Canada; 8 IRCCS Istituto Ortopedico Galeazzi, Milano, Italy; 9 Department of Physiology and Pharmacology, Gdańsk University of Physical Education & Sport, Gdańsk, Poland; 10 Vita-Salute San Raffaele University, Milano, Italy; 11 Department of Biological Sciences, California State University, Chico, CA USA; 12 Department of Stomatology, Faculty of Dentistry, Université de Montréal, Montréal, Quebec Canada

**Keywords:** Cell signalling, Mechanisms of disease

## Abstract

Adolescent idiopathic scoliosis is the most prevalent spine deformity and the molecular mechanisms underlying its pathophysiology remain poorly understood. We have previously found a differential impairment of melatonin receptor signaling in AIS osteoblasts allowing the classification of patients into three biological endophenotypes or functional groups (FG1, FG2 and FG3). Here, we provide evidence that the defect characterizing each endophenotype lies at the level of Gαi proteins leading to a systemic and generalized differential impairment of Gi-coupled receptor signaling. The three Gαi isoforms exhibited a selective serine phosphorylation patterns for each AIS endophenotype resulting in a differential reduction in Gαi protein activity as determined by cellular dielectric spectroscopy and small interfering RNA methods. We found that one endophenotype (FG2) with phosphorylated Gαi_1_ and Gαi_2_ was consistently associated with a significantly high risk of spinal deformity progression when compared to the other two endophenotypes (FG1 and FG3). We further demonstrated that each endophenotype is conserved among affected family members. This study expands our understanding of the mechanism underlying the Gi-coupled receptor signaling dysfunction occurring in AIS and provides the first evidence for its hereditary nature. Collectively, our findings offers a new perspective on Gαi hypofunctionality in a human disease by revealing specific serine phosphorylation signatures of Gαi isoforms that may facilitate the identification of AIS patients at risk of spinal deformity progression.

## Introduction

Adolescent idiopathic scoliosis (AIS) is a prevalent three-dimensional spinal deformity of unknown cause affecting an average of 2% to 4% of the pediatric population worldwide, with females affected more often in number and severity^1,2^. It is unclear why some curves progress with growth while others do not. Severe curves can present with substantial physical deformities with possible secondary cardiopulmonary problems that become life threatening when the curve exceeds a Cobb angle of 100° (the standard measure for curve magnitude)^3,4^. A dearth of information on the AIS pathophysiology has resulted in the use of the same treatment protocols that have existed for the last 30 years, i.e., observation only (for Cobb angles between 10° to 25°), external bracing (25° to 45°), and spinal surgery as a last resort (>45°)^5–7^. Early curve detection and an improved stratification of AIS patients are critical for better and effective treatment options.

AIS clinical heterogeneity is characterized by varied curve morphologies and magnitudes even among patients within the same family^8,9^. We have shown that AIS is also heterogeneous at the molecular level^10^. We demonstrated that osteoblasts of AIS patients exhibit different degrees of melatonin signaling that reduce the ability to inhibit cAMP production in response to melatonin receptor stimulation. Melatonin binds to melatonin receptors that are coupled to Gαi proteins and blocks the synthesis of cAMP produced by adenylate cyclases. We hypothesized that Gαi protein impairment could explain the melatonin-signaling pathway dysfunction^10^ and other neuroendocrine abnormalities observed in AIS^11–13^. We tested Gi-coupled receptor signaling using peripheral blood mononuclear cells (PBMCs) of AIS patients using cellular dielectric spectroscopy (CDS)^14^. The conformational changes in the G-protein-coupled receptors (GPCRs) that are activated by a signaling molecule (e.g., melatonin and iodomelatonin) are measured by CDS as impedance (Ω). Based on the CDS measurements of the differential patterns of melatonin signaling, the AIS patients were classified into three distinct biological endophenotypes or functional groups (FG1, FG2 and FG3)^10,15–17^. The CDS measurements for FG1, FG2, FG3, were 10–40 Ω, 40–80 Ω, and 80–120 Ω respectively. Melatonin signaling was normal when the CDS measurements exceeded 120 Ω^18–20^. The CDS measurements confirmed that the response of the cells of AIS patients to melatonin stimulation was mainly Gαi protein-dependent. From these observations, we concluded that we could reliably stratify the Gi signaling defects among AIS patients into three endophenotypes using melatonin receptor agonists^14^.

The objectives of the current study were to analyze the differential patterns of Gi protein coupled receptor signaling and explore their contribution in AIS pathogenesis. We investigated the clinical relevance of Gαi proteins by studying 1196 patients with AIS from two independent longitudinal cohorts. We stratified AIS patients into one of the three endophenotypes (FG1, FG2, and FG3). Osteoblasts, myoblasts, and PBMCs of a subset of the 1196 AIS patients and healthy control subjects were analyzed and we demonstrate that each AIS endophenotype exhibit a differential phosphorylation of Gαi_1_, Gαi_2,_ and Gαi_3_ isoforms at serine residues. The phosphorylation of Gαi proteins limits their signaling ability and suppresses the hormonal inhibition of cAMP production in various cell types^21–23^. Our clinical data indicate that the AIS patients classify in FG2 endophenotype were more prone to develop a severe scoliosis, contrasting significantly with AIS patients classified in FG3 or FG1 endophenotype. The present study provides the first evidence that differential Gi signaling impairment associated with each AIS endophenotype may have a hereditary component and prognostic usefulness to predict the risk of spinal deformity progression.

## Results

### Study populations

Table 1 shows characteristics of two independent longitudinal cohorts of subjects from Canada and Italy. We recruited 1310 subjects in the French-Canadian cohort consisting of 292 healthy controls without a family history of scoliosis and 1018 patients with AIS. Among control subjects, 240 were healthy controls and 52 were trauma cases. The absence of spinal deformities was confirmed in all the healthy controls through a physical exam and a longitudinal follow-up every 6 months until skeletal maturity. Of the 1018 AIS patients, 794 exhibited moderate curvatures (mean Cobb angle of 20.1° ± 9.5°) and 224 had severe curvatures (mean Cobb angle of 60.5° ± 11.9°). For the Italian replication cohort, 281 subjects were recruited consisting of 103 healthy controls without a family history of scoliosis and 178 patients with AIS (Table 1). Of the 178 AIS patients, 100 had moderate curvatures (mean Cobb angle of 19.0° ± 6.0°) and 78 were severe cases (mean Cobb angle of 58.5° ± 10.6°). The 103 control subjects assigned to this cohort were normal healthy children with neither apparent manifestations nor family history of scoliosis. Subjects of both French-Canadian and Italian cohorts were young Caucasians females aged between 10–16 years.

**Table 1:** Clinical and demographic data of the French-Canadian and Italian cohorts.

	French-Canadian Cohort (N = 1310)		Italian Cohort (N = 282)	
	N	Mean Age (years)	N	Mean Age (years)
**Control subjects**				
Healthy cases	240	12.4 ± 3.2	103	11.0 ± 1.6
Trauma cases	52	13 ± 3.1	—	—
**AIS patients**				
Moderate cases (Cobb Angle 10°− 44°)	794	13.0 ± 2.6	100	13.6 ± 2.8
Severe cases (Cobb Angle ≥ 45°)	224	15.1 ± 2.1	78	13.8 ± 2.2

### Biological endophenotype classification and clinical outcome of AIS patients

The prognostic value of our functional classification was evaluated with subjects of the French-Canadian cohort, and validated using the independent Italian cohort. To derive prognostic information from our functional classification, we examined the distribution of AIS groups among different stages of disease. For this purpose, we classified the AIS patients into one of the three AIS endophenotypes based on their impedance response measured by CDS using melatonin as agonist, as previously described^14^. The results obtained with the patients of the French-Canadian cohort are reported in Table 2 and show that 244 patients were classified in the first endophenotype (FG1), 396 in the second one (FG2), and 378 in the third endophenotype (FG3). We defined scoliosis severity as cases exhibiting a Cobb angle of 40° or more and the mild-moderate cases having a Cobb angle of 10° to 39°. We examined the distribution profiles for the three endophenotypes among the mild-moderate and severe cases of the disease, using a chi-square test for significance. We found that although FG1 endophenotype was represented equally in both disease groups (*P* = 0.545), FG3 endophenotype was overrepresented among the moderate cases (*P* = 0.0001), and FG2 endophenotype was overrepresented among the severe cases (*P* = 0.0005). Indeed, among the surgical cases, FG2 was the most prevalent entity and FG3 represented the intermediate entity, while FG1 was less frequent, with proportions of distribution of 10% for FG1, 60% for FG2 and 30% for FG3.

**Table 2:** Distribution of AIS patients in moderate and severe cases by biological endophenotype in the French-Canadian cohort.

	Moderate cases		Severe cases		Curve type		
	(Cobb Angle 10°– 44°)		(Cobb Angle ≥ 45 °)				
	N (%)	Cobb Angle	N (%)	Cobb Angle	Single	Double	Triple
					N (%)	N (%)	N (%)
FG1 (n = 244)	222 (28)	20.0 ± 10.1°	22 (10)	60.8 ± 2.4°	128 (51)	113 (45)	10 (4)
FG2 (n = 396)	262 (33)	21.5 ± 9.5°	134 (60)	60.0 ± 0.8°	186 (48)	178 (46)	23 (6)
FG3 (n = 377)	310 (39)	18.9 ± 9.4°	67 (30)	60.7 ± 2.6°	205 (54)	155 (41)	19 (5)

The results obtained with the patients of the Italian cohort show that 6 patients were classified in the first endophenotype (FG1), 88 in the second one (FG2), and 84 in the third one (FG3). Like the French-Canadian cohort, AIS patients from the Italian cohort presented also similar proportions of distribution in the mild-moderate stage, while FG2 was the most prevalent group in the severe stage with a proportion of 61% compared to 36% and 3% for FG3 and FG1, respectively (Table 3). In both cohorts, we found no relation between curve types and the endophenotypes. Proportions of patients with single, double or triple curves were similar among all endophenotypes, and triple curve was much less common than the two other curve types.

**Table 3:** Distribution of AIS patients in moderate and severe cases by biological endophenotype in the Italian cohort.

	Moderate stage		Severe stage		Curve type		
	(Cobb Angle 10–44 °)		(Cobb Angle ≥ 45 °)				
	N (%)	Cobb Angle	N (%)	Cobb Angle	Single	Double	Triple
					N (%)	N (%)	N (%)
FG1 (n = 2)	4 (4)	15.5 ± 2.5°	2 (3)	61.5 ± 5.5°	1 (50)	1 (50)	—
FG2 (n = 88)	40 (40)	24.3 ± 10°	48 (61)	53.5 ± 13.4°	37 (42)	51 (58)	—
FG3 (n = 84)	56 (56)	17.2 ± 5.7°	28 (36)	60.6 ± 12.9°	31 (37)	53 (63)	—

### Each biological endophenotype represents a potential hereditary trait

The tendency for familial clustering previously described by some authors supports the notion that AIS has a strong pattern of inheritance^9,11^. To test the possibility that the Gi-signaling defect characterizing each endophenotype may be an inherited component, patients diagnosed with AIS were interviewed with respect to their family history of spine deformities. Only patients from families with affected members within at least two generations, available to provide blood sample were considered for the family study. Eight patients from six unrelated families met this criterion. Pedigrees constructed for each of these families are shown on Supplementary Fig. S1. A minimum of two individuals were affected in each family, with at least one individual within each generation. The classification has revealed that all affected family members belonged to the same endophenotype (Table 4). Of the six families studied, one was categorized as FG1, three as FG2 and two as FG3. Clinical characteristics of affected members for whom medical records were available are shown in Table 5. It appears that at the same age, patients classified as FG2 had higher curve magnitude than patients classified as FG1 or FG3. Nevertheless, differences in curve pattern were observed in patients related or not classified into the same group. These findings suggest that progression over the time may be more heritable than expression pattern of curvature, and that each biological endophenotype co-segregates within families independently of their curve type.

**Table 4:** Functional classification and clinical data of members from studied French Canadian families.

Family ID	Case Number	Parent tie	Diagnosis	Endophenotype
Family 2	3004	Son	Scoliosis	FG1
	3005	Daughter	Scoliosis	FG1
	3006	Mother	Scoliosis	FG1
	3007	Father	—	Control
Family 11	3027	Daughter	Scoliosis	FG2
	3028	Mother	Scoliosis	FG2
	3213	Father	—	Control
Family 33	3095	Son	—	Control
	3096	Daughter	Scoliosis	FG2
	3097	Mother	Scoliosis	FG2
	3215	Father	—	Control
Family 38	3111	Son	—	Control
	3112	Daughter	Scoliosis	FG3
	3113	Mother	Scoliosis	FG3
Family 42	3123	Daughter	Scoliosis	FG3
	3124	Son	—	Control
	3125	Father	Scoliosis	FG3
	3126	Mother	—	Control
Family 73	3191	Daughter	Scoliosis	FG2
	3192	Father	—	Control
	3193	Mother	Scoliosis	FG2
	3194	Aunt	Scoliosis	FG2
	3195	Grand-mother	Scoliosis	FG2
	3196	Grand-father	—	Control

**Table 5:** Clinical characteristics of affected members from studied French-Canadian families.

Age (Years)	FG1			FG2			FG3		
	Case number	Cobb Angle	Curve pattern	Case number	Cobb Angle	Curve pattern	Case number	Cobb Angle	Curve pattern
10	3004	10°	Right thoracic	3191	10°	Left thoraco-lumbar	—	—	—
12	3006	12°	Left thoraco-lumbar	3096	12°–22°	Left Thoracic Right thoraco-lumbar	—	—	—
14	—	—	—	3095	22°	Left thoraco-lumbar	3123	16°	Left thoraco-lumbar
16	—	—	—	3027	16°	Left thoracic	3112	14°	Right thoracic

### Differential melatonin signaling responses implicate reduced Gαi protein activity in AIS

**Figure 1:**
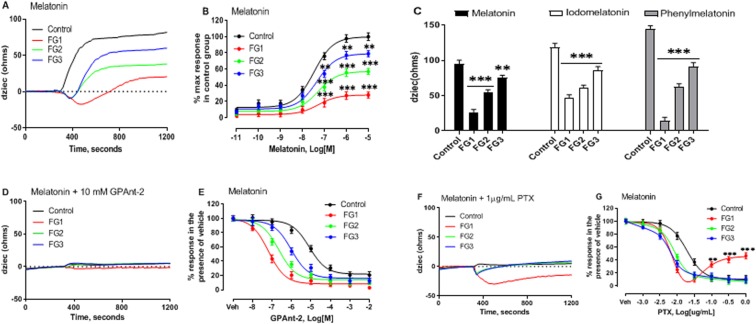
Differential melatonin signaling patterns are revealed in AIS. (**A**) The representative impedance signature generated by CDS in the control and the AIS osteoblasts following melatonin receptor stimulation. The cells were challenged with 10 µM melatonin. dZiec representing the impedance response of the cells to the electric current generated, was measured every 2 seconds. (**B**) The concentration response curves were generated with the GraphPad Prism software using data normalised to the response achieved at maximal stimulation in the control osteoblasts. (**C**) The comparative efficacy of the 3 melatonin receptor agonists on the impedance response in the control and the AIS osteoblasts. The cells were challenged with 10 µM each of the indicated compounds. **(D**) The effect of 1 h pre-treatment with 10 mM GPAnt-2 on the kinetic signature of melatonin. (**E**) The inhibition curve of GPAnt-2 in response to 10 µM melatonin plotted as a percent of the response obtained in the presence of vehicle (PBS). (**F**) The effect of 1 µg/mL PTX on the kinetic signature of melatonin following 16 h pre-treatment. (**G**) The effect of 16 h pre-treatment with varying concentrations of PTX (0.3 ng/mL, 1 ng/mL, 3 ng/mL, 10 ng/mL, 30 ng/mL, 100 ng/mL, 1 µg/mL), on the impedance response to 10 µM melatonin normalized to the response of control cells in the presence of the vehicle (PBS). Data are expressed as mean ± SEM of total number of values obtained from each of the three independent experiments for n = 12 patients per group. *P < 0.05, **P < 0.01, ***P < 0.001, versus control group based on one-way ANOVA followed by a post-hoc Dunnett’s test.

Melatonin interacts with high affinity receptors that are coupled to Gαi proteins in various cell types^24^. Keeping this in mind, we hypothesized that the defective melatonin signaling in AIS is caused by defective Gαi proteins. We used CDS to measure the impedance changes in response to melatonin, as previously described^14^, in the osteoblasts from 12 control subjects and 12 AIS patients of each of the three biological endophenotypes (FG1, FG2, FG3) previously defined. Results illustrated in Fig. 1A show that in the control osteoblasts, the impedance response rapidly increased (>120 Ω) immediately following 10 µM melatonin addition and continued in a time-dependent manner. This increase in impedance is consistent with the prototypical response of Gi-coupled receptors^18^. However, the impedance profiles generated by the AIS osteoblasts briefly decreased below zero before quickly reverting to a positive slope. The transient negative phase associated with AIS was greater in the osteoblasts of the FG1 AIS patients compared with that of the FG2 or FG3 AIS patients. These results indicate that in AIS, the response induced by melatonin receptors through their interactions with Gαi proteins is differentially modified among the three patient endophenotypes. There was also a differential decrease in the magnitude of the overall positive component of the impedance response among the three AIS endophenotypes relative to the controls (Fig. 1A).

Interestingly, the concentration-response curves at submaximal concentrations (1 μM) of melatonin between the groups were similar to those observed in Fig. 1A (Fig. 1B). The half-maximum response (EC_50_) was observed at 10 pM, 100 pM, 1 nM, 10 nM, 100 nM, 1 µM, 10 µM melatonin concentrations in the control and the AIS endophenotype groups (Supplementary Table S1), suggesting that the affinity of the melatonin receptors for their agonists is preserved in AIS cells.

Next, we examined the possibility of a change in the activity of the melatonin receptors. We compared the integrated response to melatonin in the control and the AIS osteoblasts with the response to the melatonin analogues phenylmelatonin (10 µM) and iodomelatonin (10 µM). The results illustrated in Fig. 1C show that the magnitude of the response to melatonin in the control osteoblasts and the AIS osteoblasts, reflect the efficacy grade of the agonists i.e. phenylmelatonin > iodomelatonin > melatonin^25^. The impedance responses (Ω) observed in the control group treated with phenylmelatonin, iodomelatonin, and melatonin, were 145 ± 9.43, 119 ± 5.02, and 95 ± 2.03, respectively. The percent reduction in the impedance responses of the AIS osteoblasts compared with the controls, were 90% in FG1, 57% in FG2, and 37% in FG3 for phenylmelatonin; 80% in FG1, 49% in FG2, and 28% in FG3 for iodomelatonin; and 72% in FG1, 43% in FG2, and 21% in FG3 for melatonin. These observations exclude the possibility that the defective signaling observed in AIS cells results, from either a reduced activity of the melatonin receptors or a defective coupling of the Gαi proteins.

To examine whether there is a decrease in the concentration of the functional Gαi proteins in AIS, we used the G protein antagonist GPAnt-2. This hydrophobic peptide competes with the receptors that couple to Gαi proteins and inhibits the interaction between the receptors and the Gαi proteins^26^. If the number of the functional Gαi proteins is reduced in AIS, we would expect to observe changes in the inhibitory effect of this peptide in response to melatonin in the AIS osteoblasts. Results illustrated in Fig. 1D show that GPAnt-2 eliminated the impedance changes induced by melatonin in the osteoblasts of the control subjects and the AIS patients of each biological endophenotype. The concentration-response curves revealed that this reduction was concentration-dependent (Fig. 1E). Indeed, the three AIS endophenotypes exhibited a left-shift in their concentration-response curves compared with that of the control group, and the IC_50_ values differed between the groups (Supplementary Table S2). These results suggest that the concentration of the functional Gαi proteins is reduced to different degrees among the three AIS endophenotypes.

Taking into account that GPAnt-2 competes with the receptors of the Gαi and Gαs proteins^26^, we selectively decreased the level of the functional Gαi proteins by incubating the osteoblasts with pertussis toxin (PTX). We found that the treatment with PTX not only altered the initial impedance response to melatonin in the AIS groups but also dramatically reduced the positive component in the FG1 subgroup (Fig. 1F). The concentration-response curve showed that at low concentrations of melatonin, PTX inhibited the response to melatonin in the control and the AIS groups, producing a pattern similar to the one obtained with GPAnt-2. At high concentrations, however, this treatment selectively increased the response in the FG1 endophenotype (Fig. 1G). These results demonstrate that reduced levels of functional Gαi proteins cause defects in melatonin signaling and suggest the possibility of a compensatory Gi-independent signaling pathway in cells from AIS patients classified into the FG1 endophenotype.

### Impaired Gi-mediated signaling is a generalized and systemic defect in AIS

**Figure 2:**
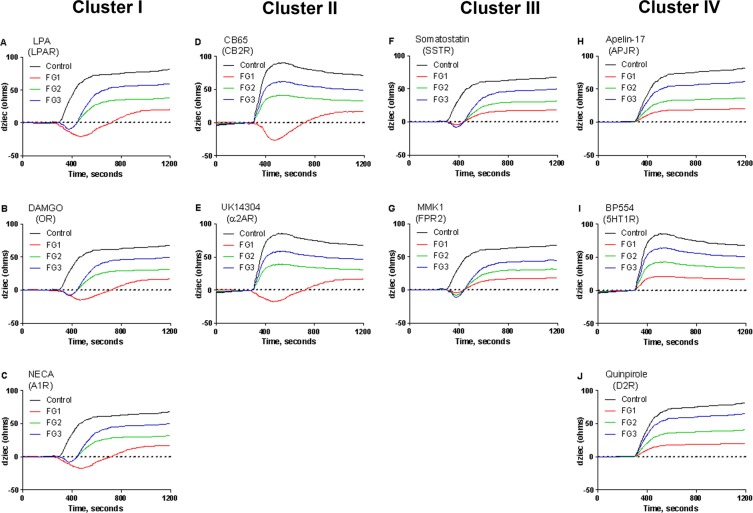
Impedance signatures of various Gi-coupled receptor agonists in AIS osteoblasts reveal four distinct clusters. Osteoblast cells were stimulated with 10 µM each of Gi-coupled receptor agonists: (**A**) LPA, (**B**) DAMGO, (**C**) NECA, (**D**) CB65, (**E**) UK14304, (**F**) Somatostatin, (**G**) MMK1, (**H**) Apelin-17, (**I**) BP554, or (**J**) Quinpirole. The targeted endogenous receptors are shown in parentheses. The impedance represented in the y-axis as dziec was measured by CDS every 2 seconds. Data are representative of the impedance signature in osteoblasts from each group of 12 individuals where duplicate values were obtained from each of the three independent experiments.

**Figure 3:**
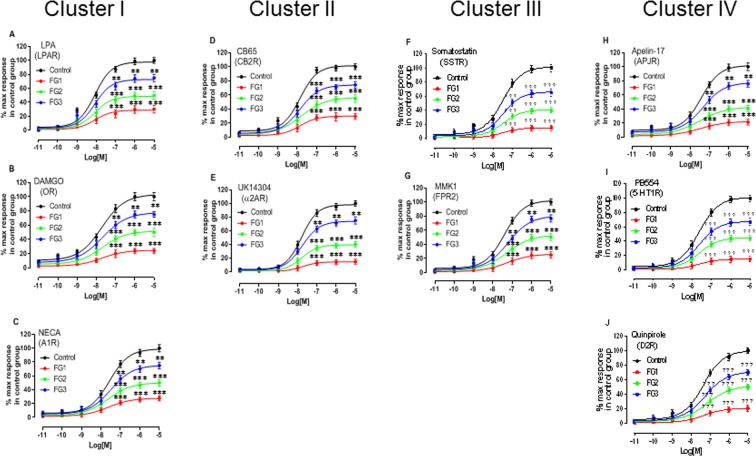
Classification of AIS patients according to their biological endophenotypes. Primary human osteoblast cultures were used to determine AIS patients’ impedance signature by cellular dielectric spectroscopy in response to various specific agonists of Gi-coupled receptors. (**A**–**J**) The agonists and targeted receptors are indicated in each panel and the concentration-response curves are generated with the GraphPad Prism software using data normalized to the response achieved at maximal stimulation in the cells from the control subjects. The data are expressed as mean ± SEM of the duplicate values obtained from each of the three independent experiments performed for n = 12 patients per group. *P < 0.05, **P < 0.01, ***P < 0.001, versus control group based on one-way ANOVA followed by a post-hoc Dunnett’s test.

To determine if the reduced ability of the Gαi proteins to promote signal transduction in AIS is restricted to melatonin receptors, we compared various synthetic compounds selectively activating other receptors coupled to Gαi proteins. Ten compounds at a concentration of 10 μM each (LPA, DAMGO, NECA, CB65, UK14304, Somatostatin, MMK1, Apelin-17, BP554, and Quinpirole), were tested, and the representative concentration-response curves for each of these compounds in the control and the AIS osteoblasts are illustrated in Fig. 2. The impedance signatures revealed that the tested compounds fell into four distinct clusters. In cluster I (Fig. 2A–C), the compounds (LPA, DAMGO, and NECA) elicit impedance profiles similar to those obtained with melatonin, consisting of a biphasic impedance shape in all the three AIS endophenotypes with a larger negative phase for the FG1 endophenotype. In cluster II (Fig. 2D,E), the compounds (CB65, UK14304) elicit a negative response only in the FG1 endophenotype. In cluster III (Fig. 2F,G), the compounds (Somatostatin, MMK1) elicit a relatively short transient negative phase to a similar extent in the three AIS endophenotypes, while in cluster IV (Fig. 2H–J), the compounds (Apelin-17, BP554, Quinpirole) totally lack this feature and elicit completely positive impedance in all the AIS endophenotypes. Despite the differences in the shapes of the impedance profiles, the regression analysis of the concentration-response curves for each tested compound revealed no significant difference in the EC_50_ values between the control and the AIS groups (Supplementary Table S1), while all the AIS endophenotypes were clearly distinguished by the amplitude of their maximum response (Fig. 3). For each compound, the three AIS endophenotypes were less responsive than the control group. The degree of reduction for each functional group relative to the control group was similar to that obtained with melatonin. This suggests that the AIS patients can be classified into one of the three biological endophenotypes using the agonists of any Gi-coupled receptor, based on the values that are established with a melatonin receptor agonist. Following the treatment with PTX at high concentrations, only receptor agonists of clusters I and II (Supplementary Fig. S2A–E) elicited the increased response in FG1 that was also observed following melatonin receptor stimulation. This further supports the notion that compensatory Gi-independent signaling occurs in the FG1 group, independent of the receptor type. In contrast, the responses to the receptor agonists of clusters III and IV were abolished in all the groups (Supplementary Fig. S2F–J). The inhibition curves of GPAnt-2 (Supplementary Fig. S3A–J) that were generated using the clustered receptor agonists revealed curve patterns similar to that obtained with melatonin receptor stimulation. In each case, GPAnt-2 caused a left-shift of the concentration-response curve in the AIS groups compared with the control group, leading to reduced EC_50_ values (Supplementary Table S2).

We examined whether this signaling defect is confined only to osteoblasts by extending our analysis to myoblasts and PBMCs. We found that both the cell types exhibited a response pattern similar to that obtained with the osteoblasts (Supplementary Figs S4 and S5).

**Figure 4:**
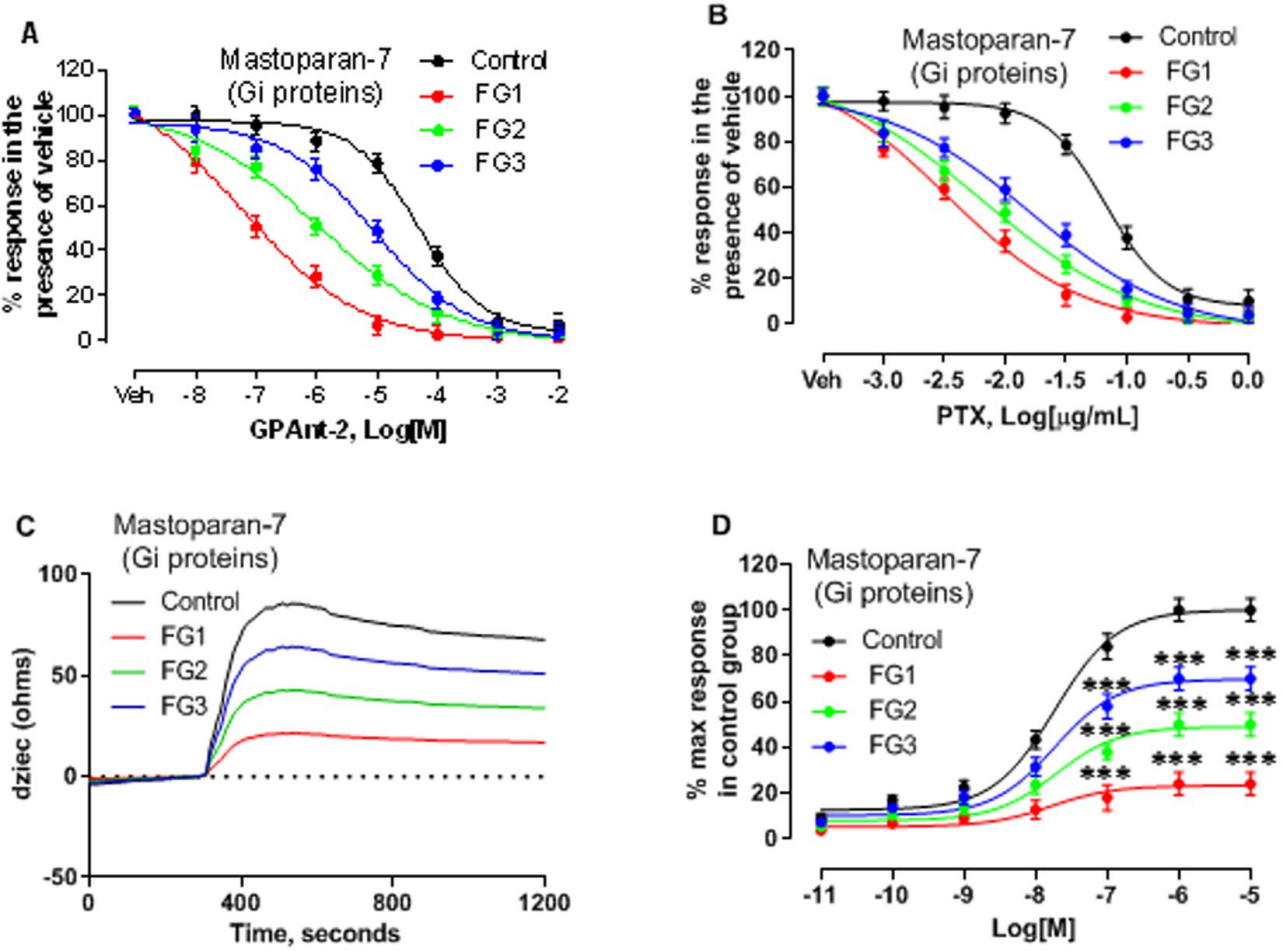
Reduced Gαi protein activity among the AIS groups is revealed by direct activation of the Gi proteins with mastoparan-7. The inhibition curves for the response to 10 µM mastoparan-7 following (**A**) 1 h treatment of (10 mM) GPAnt-2 and (**B**) 16 h treatment of 1 µg/mL PTX. Data within each group were normalized to the response in the presence of the vehicle (PBS). (**C**) Representative impedance signatures of mastoparan-7 in the control and the AIS osteoblasts. The cells were challenged with 10 µM mastoparan-7. dZiec representing the impedance response to the electric currents generated in the cells, was measured every 2 sec. (**D**) The concentration response curves (10 pM, 100 pM, 1 nM, 10 nM, 100 nM, 1 µM, 10 µM) of mastoparan were generated with the GraphPad Prism software using data normalised to the response at maximal stimulation in cells from the control subjects. The data are expressed as mean ± SEM of the values where duplicate values were obtained from each of the three independent experiments for n = 12 patients per group. *P < 0.05, **P < 0.01, ***P < 0.001, versus control group based on one-way ANOVA followed by a post-hoc Dunnett’s test.

Similar responses were observed when mastoparan-7 competes with GPAnt-2 (Fig. 4A) and directly activates Gαi proteins by mimicking an agonist-activated receptor^27,28^. Interestingly, the response to mastoparan-7 was almost abolished in all the AIS and control groups, following treatment with high concentrations of PTX (Fig. 4B), suggesting an abnormality in the level of the Gαi proteins. Although mastoparan-7 produced Gαi protein-like profiles in the control and the AIS groups (Fig. 4C), the impedance responses were much lower in the AIS groups compared with the control group (Fig. 4C,D). These data indicate that the defective Gi-mediated signaling in AIS is due to reduced Gαi protein activity and represents possibly a generalized impairment in AIS.

### Reduced Gαi protein activity in AIS favors the alternate coupling of Gαs proteins

**Figure 5:**
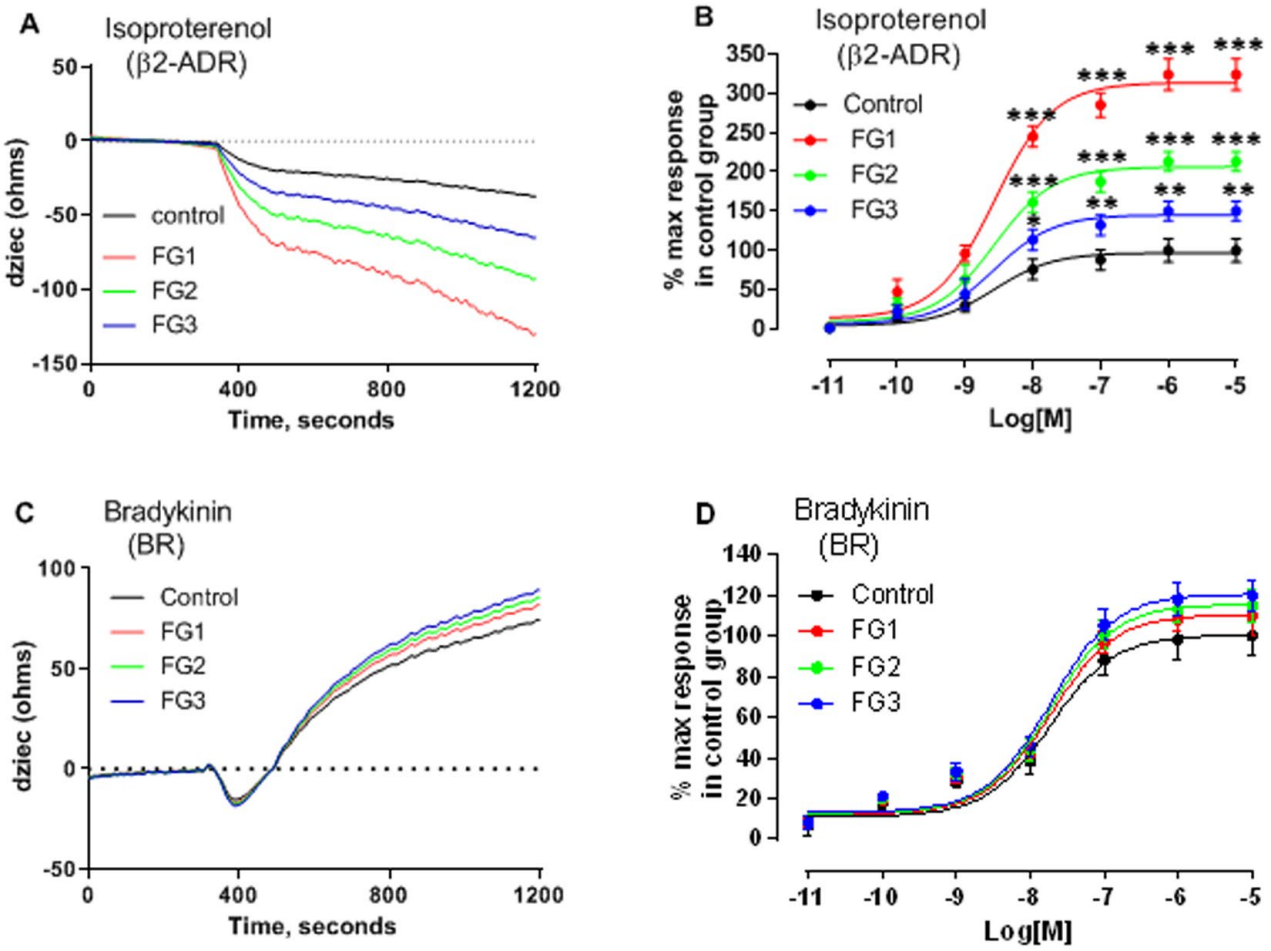
Functional status of the Gαs and Gαq proteins in the osteoblasts from the control and the AIS functional groups. The functionality of the Gαs and Gαq proteins was evaluated by challenging the cells with (**A**,**B**) isoproterenol (10 μM) or (**C**,**D**) bradykinin (10 μM), respectively. Representative impedance signatures generated by the CellKey^TM^ system in the control and AIS osteoblasts in response to 10 µM of (**A**) isoproterenol or (**C**) bradykinin acting through their cognate receptors are shown in parentheses. The impedance response represented in the y-axis as dziec was measured every 2 sec. The concentration response curves (10 pM, 100 pM, 1 nM, 10 nM, 100 nM, 1 µM, 10 µM) of (**B**) isoproterenol or (**D**) bradykinin were generated with the GraphPad Prism software using data normalized to the response of maximal stimulation of cells from the control subjects. The data are expressed as mean ± SEM of values obtained from each of the three independent experiments for n = 12 patients per group. *P < 0.05, **P < 0.01, ***P < 0.001, versus control group based on one-way ANOVA followed by a post-hoc Dunnett’s test.

The diversity in the impedance profiles of GPCR signaling is currently limited to the three standard Gαs, Gαi, and Gαq proteins^18^. Since many receptors coupled to the Gαi proteins can also couple with the Gαs and/or the Gαq proteins, we hypothesized that the biphasic impedance profiles observed in the AIS cells may be from the dual coupling of the Gi-coupled receptors to the Gαs or Gαq proteins. To test this hypothesis, we first examined the functional status of the Gαs and the Gαq proteins. The osteoblasts from the control and the AIS patients were screened for their response to 10 μM isoproterenol and bradykinin, which activate Gαs and Gαq proteins respectively. The results illustrated in Fig. 5A show that isoproterenol elicited a qualitatively similar impedance signature in the control and the AIS osteoblasts, consisting of a decrease in the impedance response. However, although the shape of their impedance profiles was similar, the magnitude of the response in the AIS cells was higher than that of the control group and differed among the AIS endophenotypes (Fig. 5A,B). Similarly, the impedance response to bradykinin stimulation also maintained the same general shape in the control and the AIS osteoblasts, consisting of positive impedance after a transient dip (Fig. 5C). In contrast to isoproterenol, the maximum impedance response elicited by bradykinin was similar in the control and the AIS osteoblasts (Fig. 5C,D). These results indicate that the activity of the Gαs proteins is increased in AIS due to the reduced Gαi protein function associated with this disease. The concentration-response curve in the control and the AIS osteoblasts revealed that the magnitude of the response to Gαs stimulation inversely mirrored the magnitude of the response to Gαi stimulation.

We next examined the possibility that the difference in the impedance shape among the AIS groups in response to Gi-coupled receptor activation was due to the Gαs or Gαq proteins. For this purpose, we used small interference RNA (siRNA) to knock down the Gαs or Gαq expression before stimulating the cells. Quantitative real-time PCR (qRT-PCR) and Western blot analyses confirmed the efficiency of the siRNA approach (Supplementary Fig. S6A,B). The results illustrated in Supplementary Fig. S6 show that Gαs or Gαq protein depletion has no effect on the impedance signature of any of the Gi-coupled receptor agonists in the control group (Supplementary Fig. S6A–D). In contrast, in the FG1 endophenotype (Supplementary Fig. S6E–H), the negative phase was completely abrogated by the depletion of Gαs proteins for clusters I and II and by the deletion of Gαq proteins for cluster III, while the cluster IV that exhibited monophasic impedance shape remained unchanged. Interestingly, the positive phase was not affected by the deletions in all four clusters. In the FG2 endophenotype (Supplementary Fig. S6I–L), the depletion of the Gαs protein had no effect on the negative phase in any of the clusters, while the reduction of the Gαq protein led to the loss of the negative phase without affecting the positive phase. Similar observations were noticed in FG3 endophenotype (Supplementary Fig. S6M–P). These results suggest that Gαs and Gαq protein-dependent responses are integrated in the biphasic impedance signature of the Gi-coupled receptors in AIS. Our impedance results are consistent with previous cAMP accumulation in AIS osteoblasts^10,14–17^ upon the activation of melatonin receptors, which indicates a Gαs-dependent protein response in FG1 endophenotype because of the lack of functional Gαi proteins.

**Figure 6:**
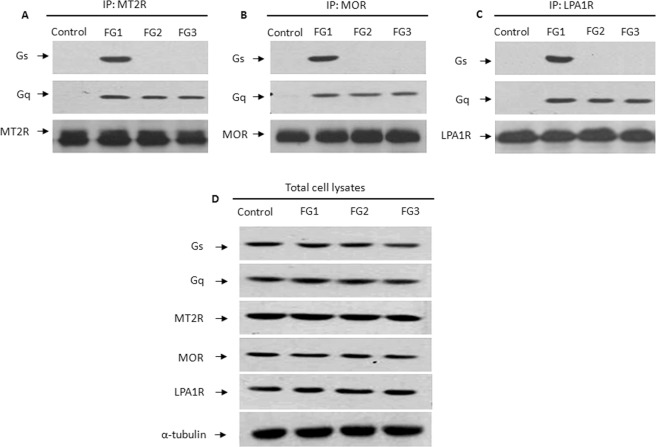
Selective dual interaction of Gi-coupled receptors with Gαs and Gαq proteins is revealed in FG1. Whole osteoblasts from the control subjects and the AIS patients of each functional group were subjected to immunoprecipitation with antibodies to (**A**) the melatonin receptor (MT2R), (**B**) the µ-Opioid receptor (MOR), or (**C**) the LPA type 1 receptor (LPA1R). The precipitates were resolved by 10% SDS-PAGE and immunoblotted with the Gαs or Gαq specific antibody. The precipitates were also analyzed with antibodies against the indicated receptors. (**D**) The expression of Gαs, Gαq, and the three receptors in each group, was confirmed by western blot analysis of the total cell lysates. The bands shown are representative of the results obtained with the osteoblasts from the 12 different patients of each group. *The images are a representation of the original blots and full-length blots are presented in Supplementary Fig. S11.* *Each strip originates from a distinct original gel and is stacked for illustration purposes. Experiments were run in parallel and under the same experimental conditions*.

To further assess the specificity of Gi-coupled receptor coupling to the Gαs protein in FG1 endophenotype, three different Gi-coupled receptors [(melatonin receptor (MT2R), µ-opioid receptor (MOR), and LPA type 1 receptor (LPA1R)] were immunoprecipitated in the osteoblasts from the control subjects or the patients of each endophenotype group and blotted with an antibody against the Gαs or Gαq protein. As expected, we found that the Gαs protein was present in the precipitates of each of these receptors in the FG1 osteoblasts but not in the FG2 or FG3 cells, while Gαq was precipitated with the three receptors in all the AIS endophenotypes. The precipitates of each of these receptors contain neither Gαs nor Gαq proteins in the control group (Fig. 6A,C). Western blot analysis confirmed the presence of these proteins in equal amounts in the control and the AIS endophenotypes (Fig. 6D).

### Differential phosphorylation of Gαi protein isoforms among AIS endophenotypes

Phosphorylation of the Gαi proteins limits their ability to transduce signals^21,23,29–34^. We previously reported an increased phosphorylation of the serine residues of the Gαi_1_, Gαi_2_, and Gαi_3_ isoforms of the Gαi proteins in the osteoblasts from AIS patients^10^. Given that all the Gαi isoforms share the same properties in various cell types and that some isoforms are more efficient than others are, we hypothesized that the divergence in the response characterizing the three AIS endophenotypes is due to a structural modification of the three indicated Gαi protein isoforms. To address this hypothesis, we first examined the gene and protein expression levels of these isoforms as well as the expression of the Gαs and Gαq proteins in the osteoblasts from the control and the AIS patients. The qRT-PCR analysis revealed no significant change in the mRNA expression between the control and the AIS osteoblasts for the three isoforms of the Gαi proteins (Gαi_1_, Gαi_2_ and Gαi_3_) as well as the Gαs or Gαq proteins (Supplementary Fig. S7A). Assuming equal amplification of the different Gαi isoform mRNAs, it appears that Gαi_1_ and Gαi_2_ are the most abundant Gαi protein isoforms in the control and the AIS osteoblasts, while the expression level of the Gαi_3_ isoform is similar to that of the Gαs protein. The level of the Gαi_1_, Gαi_2_, Gαi_3_, Gαs, and Gαq proteins detected in the AIS osteoblasts were similar to those found in the control osteoblasts (Supplementary Fig. S7B). These results indicate that the differences in Gi-mediated signaling among the AIS groups were not due to the differences in the expression of the Gαi protein isoforms or the other G proteins.

**Figure 7:**
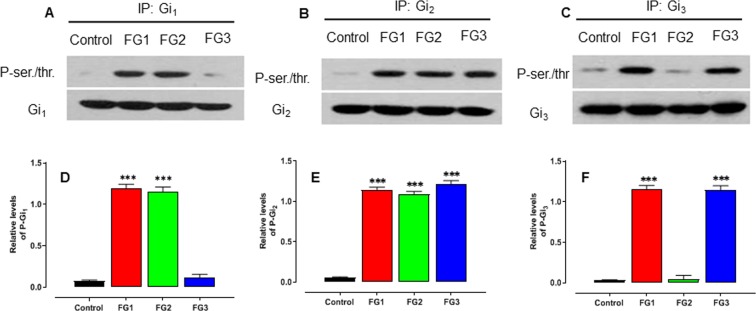
Differential phosphorylation patterns of Gαi protein isoforms in the AIS functional groups. Whole osteoblast cells from the control subjects or the AIS patients were subjected to immunoprecipitation with antibodies to (**A**) Gαi_1_, (**B**) Gαi_2_, or (**C**) Gαi_3_. The precipitates were resolved by 10% SDS-PAGE and immunoblotted with antibodies directed against phospho-serine/threonine or the indicated G protein isoform. The bands shown are representative of the results. (**D-F**) The histograms indicate the relative level of the corresponding phosphorylated Gαi protein isoform (Supplementary Table S5). Error bars show SEM for n = 12 patients per group. *P < 0.05, **P < 0.01, ***P < 0.001, versus control group based on one-way ANOVA followed by a post-hoc Dunnett’s test. *The images are a representation of the original blots and full-length blots are presented in Supplementary Fig. S12.* *Each strip originates from a distinct original gel, and is stacked for illustration purposes. Experiments were run in parallel and under the same experimental conditions*.

To examine whether there is a difference in the phosphorylation status of the Gαi protein isoforms among the AIS groups, the Gαi_1_, Gαi_2,_ and Gαi_3_ isoforms were immunoprecipitated in the osteoblasts from the control subjects and the AIS patients of each endophenotype, and then probed with an anti-phosphoserine antibody. Compared with the control group, we found an increased phosphorylation of the Gαi_1_ isoform in FG1 and FG2 osteoblasts but not in FG3 osteoblasts. The Gαi_2_ isoform was phosphorylated in all the three AIS endophenotypes, and only FG1 and FG3 osteoblasts exhibited a phosphorylation of the Gαi_3_ isoform (Fig. 7). These results indicate that the heterogeneous nature of the defective Gi-mediated signaling associated with AIS involves a selective phosphorylation of the Gαi protein isoforms.

To test this hypothesis, we used siRNA to knock down either individually or in combination, the expression of Gαi_1_, Gαi_2_, Gαi_3_, Gαs, and Gαq proteins. The silencing of each gene reduced the expression of the corresponding mRNA in the osteoblasts of the control and the three AIS endophenotypes by 75% to 85% (Supplementary Fig. S8A). Similar results were obtained at the protein level (Supplementary Fig. S8B). We also validated the selectivity of the siRNA for each entity.

Since many receptors couple to only certain Gαi isoforms, we chose to stimulate cells with melatonin or LPA to activate their cognate receptors that were shown to couple to Gαi_1_, Gαi_2_, and Gαi_3_ protein isoforms. The results illustrated in Supplementary Fig. S9 show that when Gαi_1_, Gαi_2_, or Gαi_3_ siRNA were transfected alone, the response of the control group to both agonists was not significantly affected, but was almost abolished when the siRNAs were transfected together. However, the selective depletion of Gαs or Gαq proteins was devoid of any effect. In the FG1 endophenotype, the response to melatonin or LPA was not affected by the knock down of the individual Gαi isoforms, but was reduced by 50% following the depletion of all three Gαi isoforms. The silencing of Gαs alone reduced the response to the two tested compounds by 50%, while the selective depletion of the Gαq protein had no effect in this AIS endophenotype. In the FG2 endophenotype, the response to each tested agonist was reduced by at least 75% after silencing the Gαi_3_ isoform alone but was not significantly affected when the Gαi_1_, Gαi_2_, Gαs, or Gαq proteins were selectively depleted. Likewise, in the FG3 endophenotype, only the selective depletion of the Gαi_1_ isoform caused a significant reduction in the response to the tested compounds. Individual depletion of the other Gαi isoforms as well as the Gαs or Gαq proteins did not have any effect in the FG3 osteoblasts. Our results identify Gαi_3_ and Gαi_1_ as the isoforms responsible for the residual response to Gi-coupled receptor stimulation in FG2 and FG3 endophenotype respectively, and indicate that the response in FG1 is an additive effect of these receptors where both tested Gi-coupled receptors (melatonin and LPA receptors) couple simultaneously to the Gαs and Gαi proteins.

## Discussion

AIS is a common, complex multifactorial disease and one of the most prevalent childhood deformities worldwide^11^. On average, AIS affects 2–4% of the global pediatric population^11^. It represents a serious and chronic health condition affecting individuals throughout their lives. More than seven million patients in the US are diagnosed with scoliosis (over 350,000 in Canada). Most are diagnosed with scoliosis between the ages of 10 and 15, and one out of every six children will have a progressive curve that requires active treatment. Efforts to identify genomic variants that may predict the risk of spinal deformity progression have generated many variants with minimal effects (reviewed in^35^). Using multiple cell types (osteoblasts, myoblasts and PBMCs) from AIS patients and healthy controls, we identified, for the first time, a common disease mechanism among all the AIS patients based on a differential impairment of Gi-coupled receptor signaling activities, which appears to be systemic and generalized. Such Gi-signaling dysfunction led to the classification of AIS patients into three biological endophenotypes (FG1, FG2 and FG3) based on the selective serine phosphorylation of one or more Gαi isoforms (Gαi_1_, Gαi_2_ and Gαi_3_). As previously defined, these endophenotypes are representative of the genes and molecular pathways underlying AIS pathogenesis^36,37^. By studying two independent longitudinal cohorts in distinct Caucasian populations, we showed that AIS patients classified in FG2 endophenotype develop more often a severe scoliosis (Cobb angle > 45°) requiring a surgical treatment when compared to FG1 and FG3 endophenotype.

Indeed, we observed that all the Gαi isoforms were phosphorylated in the FG1 osteoblasts, whereas only Gαi_1_ and Gαi_2_ were phosphorylated in FG2 osteoblasts, and Gαi_2_ and Gαi_3_ were phosphorylated in FG3 osteoblasts. The selective depletion of the Gαi_3_ and Gαi_1_ isoforms in the FG2 and FG3 osteoblasts respectively almost abolished their cellular responses toward three distinct Gi-coupled receptors in a similar manner, further confirming that Gαi_3_ and Gαi_1_ achieve the residual Gi signaling in FG2 and FG3 osteoblasts respectively. This is consistent with the concept that each Gαi isoform can partially rescue Gi-coupled receptor signaling. Nevertheless, FG2 osteoblasts exhibited a weaker residual Gi signaling when compared with FG3 osteoblasts, which further support our previous data obtained with cAMP assays showing that FG2 osteoblasts were almost unable to inhibit cAMP production induced by forskolin stimulation^10,15^. This is in agreement with several reports indicating that Gαi_3_ is less efficient to inhibit cAMP production when compared with the other Gαi isoforms^38–40^ and the fact that Gαi_3_ isoform is less abundant in human osteoblasts, as demonstrated in the present study. Of note, depletion of all three isoforms by siRNA interference almost abrogated the signaling impedance in FG2 and FG3 osteoblasts, contrasting with FG1 osteoblasts where 50% of Gαi signaling activity was maintained despite the fact that all three isoforms were phosphorylated. These results suggest a possible heterogeneity in the number and position of serine residues phosphorylated among Gαi isoforms in each AIS endophenotype.

Given that GPCRs promiscuously couple to and activate signaling through multiple G proteins^41^, we observed that the reduced level of the functional Gαi proteins enhances the ability of their cognate receptor to couple to Gαs or Gαq proteins in AIS. However, the magnitude of the response was not affected when Gαq proteins are depleted; eliminating the possibility, that Gαq signaling is acting as a compensatory signaling pathway when Gαi proteins are impaired in AIS. In contrast, depletion of the Gαs protein results in a significant reduction in the magnitude of the Gi signaling response in AIS osteoblasts. Indeed, several reports indicate that the inhibition of the Gαi proteins enhances Gs-coupled receptor signaling^21,22,42–45^. Our results reveal a similar relationship in AIS in particular with the FG1 endophenotype where the phosphorylation of the three Gαi isoforms results in the permissive coupling of Gαs with three different Gi-coupled receptors (MT2R, MOR and LPA1R) as demonstrated by our co-immunoprecipitation assays. On a clinical point of view, our data are potentially interesting for the postsurgical pain management of scoliotic patients classified in FG1 endophenotype because scoliosis surgery is associated with considerable postoperative pain requiring more analgesia when compared for other major surgical procedures. Indeed, the uncoupling of Gαi proteins from μ-opioid receptors is one of the known mechanisms underlying morphine tolerance^46,47^. Normally, the activation of μ receptor by morphine and subsequent Gαi protein coupling results in the inhibition of adenylyl cyclase activity and the interruption of nociceptive signal^48,49^. Repeated administration of morphine causes a switch from Gαi protein to Gαs protein coupled to μ receptors causing their activation and leading to morphine tolerance and reduced analgesia^50,51^. The pre-existing mechanism underlying the switch from Gαi protein to Gαs protein in AIS cells in absence of morphine is not exclusive to μ receptors since MT2R and LPA1R are similarly affected. Therefore, it is conceivable that differential morphine tolerance and morphine-induced hyperalgesia (increased pain with escalating doses) could potentially complicate the postoperative management of AIS patients classified in FG1 endophenotype.

The hypofunctionality of Gi proteins was previously reported in patients suffering from fibromyalgia (FM)^45^, migraine with aura (MA), or migraine without aura (MO)^52^. Interestingly, X-ray examination revealed that 80.6% of the patients with FM exhibit scoliosis, contrasting with only 8.9% of the age-matched control population^53^. Furthermore, Brox *et al*., reported that migraine was the second most common comorbidity in middle-aged patients with idiopathic scoliosis^54^. More interestingly, the Gi protein hypofunctionality in FM patients and those with MO exhibit a cAMP-inhibitory curve response in the presence of Gpp(NH)p similar to the one obtained with FG2 AIS patients^10,52^. Of note, patients suffering from MA exhibit, in the same conditions, a cAMP-inhibitory curve response similar to the one obtained with AIS patients classified in the FG1 endophenotype^10,52^.

In the present study, we acknowledge some limitations. First, this study was not designed to determine the nature of the phosphorylated serine residues among Gαi protein isoforms tested. Our attempts by distinct phospho-proteomic approaches failed to revealed specific phospho-peptides among the endogenous Gαi isoforms, which could explain why most (if not all) previous studies used indirect *in vitro* methods with either recombinant proteins or transfected cells overexpressing wild-type or mutated Gαi isoforms. Clearly, this is not a trivial task and much more work is required to determine the molecular mechanisms underlying the selective phosphorylation of endogenous Gαi isoforms in AIS. Secondly, there is a need for validation of these endophenotypes in other replication cohorts and in different ethnic groups affected by AIS as well as in other diseases, like FM, where scoliosis is a frequent comorbidity.

In summary, Gαi protein hypofunctionality may represent a novel and unrecognized molecular mechanism underlying AIS pathogenesis and may explain several endocrine disturbances occurring in AIS as well as the higher prevalence of spinal deformity progression in patients classified in the FG2 endophenotype.

## Materials and Methods

### Study population

A total of 1591 subjects were enrolled in two independent studies in Montreal (Canada) and Milan (Italy). For the first cohort (French-Canadians, Montreal), 1018 AIS patients were recruited consecutively between 2008 and 2013 from The Sainte-Justine University Hospital, The Montreal Children’s Hospital, and The Shriners Hospital for Children, in Canada (Table 1). Fifty-two children undergoing trauma surgery in collaborating hospitals and 240 healthy children recruited randomly from local schools were used as controls (Table 1). Of the 1018 AIS patients, 794 exhibited moderate curvatures (mean Cobb angle of 20.13 ± 9.5°) and 224 had severe curvatures (mean Cobb angle of 60.5 ± 11.9°) at their last visit. For the Italian replication cohort, 178 AIS patients were recruited consecutively between 2008 and 2014 from The IRCCS Istituto Ortopedico Galeazzi, a leading private orthopedic hospital and research institute in Milan (Table 3). Of the 178 AIS subjects, 100 had moderate curvatures (mean Cobb angle of 19 ± 6°) and 78 were severe cases (mean Cobb angle of 58.5 ± 10.6°). One hundred three healthy children were recruited randomly from local schools in Milan using the same criteria as indicated above. The 103 control subjects were normal healthy children with neither apparent manifestations nor a family history of scoliosis. The subjects of both the French-Canadian and the Italian cohorts were young Caucasian females aged between 10 and 16 years. The diagnosis of AIS was established by clinical and radiological examinations, and the curve magnitude was evaluated by measuring the Cobb angle at the coronal plane of the whole spine on the radiographic film. For patients with multiple curves, the magnitude of the largest curve was used for analysis. Eight hundred ninety-four patients with a Cobb angle between 10° to 39° were considered as moderate cases, and 302 patients with a Cobb angle ≥ 40° were designated as severe cases. The control subjects were also physically examined by one of the orthopedic surgeons associated with this study (H.L., J.-M.M.-T., G.G., J.O., S.P., C-H.R. and M.B.-B.) to rule out any form of scoliosis before enrolment. All the subjects were interviewed to know whether they have a family history of scoliosis. The subjects with a history of secondary or syndromic scoliosis were excluded from the study.

### Ethics statement

Informed written consent was obtained from the parents or the legal guardians of all participants, and minors gave their assent. The study in Montreal (Canada) was approved by the Institutional Review Board (IRB) of Sainte-Justine University Hospital, The Montreal Children’s Hospital, The Shriners Hospital for Children and McGill University as well as by The Affluent School Board and The English School Board of Montreal. The study in Milan (Italy) was approved by the IRB ASL Città di Milano and Istituto Ortopedico Galeazzi. All aspects of this research were performed in accordance with the relevant guidelines and regulations.

### Isolation of human osteoblasts, myoblasts, and PBMCs

The osteoblasts were prepared, as previously described^10^, from the vertebral bone specimens of 36 AIS patients undergoing spine surgery and from the tibia or femur of 12 non-scoliotic patients subjected to surgery to treat a traumatic condition. The myoblasts were isolated from the skeletal muscle specimens obtained intraoperatively from 36 AIS patients and 12 non-scoliotic patients that were subjected to surgery. Each skeletal muscle specimen was cleared of fatty and connective tissue prior to being cut into small pieces. The tissues were then transferred to a phosphate-buffered saline (PBS) solution containing 0.01% collagenase, and digested for 45 min at 37 °C. After diluting in alpha-MEM (1:1), the solution was filtered through a 45 µm nylon filter and centrifuged at 280 x g for 5 min at room temperature. The pellet containing myoblasts was suspended in culture media (alpha-MEM) supplemented with 20% fetal bovine serum (certified FBS; Invitrogen Life technologies, ON, Canada) and 1% penicillin/streptomycin (Invitrogen Life Technologies, ON, Canada). After two weeks, the culture media was replaced by fresh culture media supplemented with 10% FBS and 1% penicillin/streptomycin, and the myoblasts were allowed to grow to confluence. All the subjects were asked to provide a blood sample. Peripheral blood mononuclear cells (PBMC) were isolated from the whole blood of 36 AIS patients and 12 non-scoliotic patients by density gradient centrifugation (Ficoll-Hypaque; GE Healthcare, UK), following the manufacturer’s standard procedure.

### Functional evaluation of G protein signal transduction

The functionality of the Gαi, Gαs, and Gαq proteins was evaluated with the CDS assay, as previously described, in osteoblasts, myoblasts, and PBMCs of the AIS patients and the normal controls^11^. The GPCR agonists melatonin, iodomelatonin, phenylmelatonin, lysophosphatidic acid (LPA), the selective μ-opioid agonist D-Ala2, NMe-Phe4, Glyol5-enkephalin (DAMGO), adenosine A1 receptor (NECA), type 2 cannabinoid receptor (CB65), a2 adrenergic receptor (UK14304), Somatostatin, MMK1, Apelin-17, BP554, and Quinpirole, used in the study at a concentration of 10 μM each, were from Tocris Bioscience Bio-Techne (MN, USA). Melatonin was also used at a submaximal concentration of 1 μM. The patients were classified, as previously described, and based on the decreased impedance response to melatonin receptor stimulation compared with the control subjects^11^. Similarly the impedance responses to the activator - GpAnt-2 (Tocris Bioscience, MN, USA), the competitor – mastoparan (Tocris Bioscience, MN, USA), and the inhibitor – PTX (Sigma-Aldrich, ON, CA) were analyzed.

### RNA interference

All Gαi_1_, Gαi_2_, Gαi_3_, Gαs, Gαq, and scrambled siRNA (10 μM each) were obtained from Ambion (Ambion® Thermo Fisher Scientific, NY, USA). The sequences used for gene silencing are shown in Supplementary Table S3. Osteoblasts from the control subjects and the AIS patients were transiently transfected in serum-free medium, using the Lipofectamine RNAiMAX reagent (Invitrogen Life Technologies, ON, Canada), based on the manufacturer’s instructions. Approximately 80,000 cells were seeded at 60–80% in 96-well microplates the day prior to transfection. Lipofectamine RNAiMAX reagent as well as negative controls and siRNAs were first diluted in OPTI-MEM media (1:33.3). Each mixture was then incubated at room temperature for 5 minutes. Diluted Lipofectamine reagent was then mixed with siRNA and control samples (1:1). The mixtures were then incubated once more at room temperature for 20 minutes. The siRNA/reagent mix was then added to the microplate (20 μl/well) and the plate was incubated at 37 °C, 5% CO_2_. Functional experiments were performed 48 h post transfection. The gene knockdown was evaluated by qRT-PCR.

### Quantitative real-time PCR

RNA was isolated from the osteoblasts using TRIzol reagent (Invitrogen Life Technologies, ON, Canada) based on the manufacturer’s protocol. The total RNA (1 µg) was reverse-transcribed into cDNA using the Tetro cDNA synthesis Kit (Bioline USA Inc., MA, USA). Following cDNA synthesis, qRT-PCR was performed using a PCR master mix containing the QuantiTect SYBR Green PCR Master Mix 2 × (7 μl in 20 μl total reaction volume) (QIAGEN Inc., ON, Canada). The amplification consisted of 45 cycles of the following program: denaturation at 95 °C for 195 s; annealing at 60 °C for 15 s, extension at 72 °C for 15 s and dissociation at 95 °C for 15 s, 60 °C for 15 s and 90 °C for 15 s. The transcript expression was assessed with Stratagene Mx3000P (Agilent Technologies, CA, USA), and the calculations were performed according to the ΔΔCT method using β-actin as an internal control. The sequences of the forward and reverse primers used to identify the mRNA of the Gαi_1_, Gαi_2_, Gαi_3_, Gαs, and Gαq genes, are shown in Supplementary Table S4.

### Immunoprecipitation and western blot analyses

Osteoblasts from the AIS patients and the trauma control cases were lysed in RIPA buffer (25 mM Tris.HCl pH 7.4, 150 mM NaCl, 1% NP-40, 1% sodium deoxycholate, 0.1% SDS) containing 5 mM NaVO_4_ and the protease inhibitor cocktail (Roche Molecular Biochemicals, Mannheim, Germany). The immunoprecipitations (IP) were carried out with 1 mg of the total protein extract. The lysates were first pre-cleared with protein G beads for 1 h. The supernatant was then incubated with antibodies directed specifically against the melatonin receptor (1:1000 dilution; MEL-1B-R(G-20); goat polyclonal; sc28453; Santa Cruz Biotechnology, Inc., TX, USA), the mu-opioid receptor (1:1000 dilution; anti-Mu opioid receptor antibody; rabbit monoclonal; ab134054; Abcam, Toronto, CA), the LPA receptor (1:1000 dilution; anti-EDG2; rabbit monoclonal; ab166903; Abcam®, ON, Canada), or phosphoserine/threonine α-tubulin (1:1000 dilution; anti-phosphoserine/threonine antibody; rabbit polyclonal; ab17464; Abcam®, ON, CA), followed by a 1 h incubation with protein G beads with gentle rocking. The beads were washed 3 times with RIPA lysis buffer and the bound proteins were eluted with a Western blot loading buffer 2X and boiled at 100 °C for 5 minutes, before being separated on a 10% SDS-PAGE gel and blotted onto nitrocellulose. The blots were then examined using antibodies directed specifically against Gi_1_, Gi_2_, Gi_3_, Gs, or Gq [Gαi-1 antibody (R4) - mouse monoclonal (sc13533); Gαi-2 antibody (T-19) - rabbit polyclonal (sc7276); Gαi-3 antibody (C-10) - rabbit polyclonal (sc262); Gαs antibody (K-20) - rabbit polyclonal (sc823); Gαq/11 antibody (C-19) - rabbit polyclonal (sc392)]. All the antibodies were used at a 1:1000 dilution and were purchased at Santa Cruz Biotechnology. The protein lysates were also subjected directly to Western blot analyses with specific antibodies to the melatonin receptor, mu-opioid receptor, LPA receptor, or α-tubulin (1:4000 dilution; anti-α-tubulin antibody; mouse monoclonal clone B-5-1-2; T5168; Sigma-Aldrich Co. LLC., MO, USA). The bands were visualized using the SuperSignal chemiluminescent substrate (Thermo Fisher Scientific Inc., NY, USA).

### Statistics

Data reported represent mean values ± SEM. The statistical analysis was performed by multiple comparisons of means with the GraphPad Prism 5.0 software (GraphPad Software) using one-way ANOVA followed by a post-hoc Dunnett’s test. Only P values < 0.05 were considered significant. The clinical distributions were compared using a chi-square test.

## Supplementary information

Supplemental Information
