# Supplementary material for: A Differential Hypofunctionality of Gαi Proteins Occurs in Adolescent Idiopathic Scoliosis and Correlates with the Risk of Disease Progression
Source: Sci Rep. 2019 Jul 11;9:10074. doi: 10.1038/s41598-019-46325-2 (PMC6624302; doi:10.1038/s41598-019-46325-2)
Supplement: Supplementary file 1 — Supplemental Information [file 41598_2019_46325_MOESM1_ESM.pdf]

A Differential Hypofunctionality of Gi proteins Occurs in Adolescent Idiopathic Scoliosis and Correlates with the Risk of Disease Progression

Marie-Yvonne Akoume,[1,2] Mohamed Elbakry,[1,3,4] Maxime Veillette,[1] Anita Franco,[1] Dina Nada,[1] Hubert Labelle,[5,6] Jean-Marc Mac-Thiong,[6] Guy Grimard,[6] Jean Ouellet,[7] Stefan Parent,[6] Charles-Hilaire Rivard,[6] Giovanni Lombardi,[8,9] Alessandra Colombini,[8] Giuseppe Banfi,[8,10] Marco Brayda-Bruno,[8] Kristen F Gorman,[1,11] Alain Moreau[1,4,12,*]

[1] Viscogliosi Laboratory in Molecular Genetics of Musculoskeletal Diseases, Sainte-Justine University Hospital Research Center, Montréal, Quebec, Canada.

[2] Department of Pharmacology and Toxicology, Faculty of Medicine, Université des Sciences de la Santé (USS) de Libreville, Libreville, Gabon.

[3] Biochemistry section, Chemistry Department, Faculty of Science, Tanta University, Egypt.

[4] Department of Biochemistry and Molecular Medicine, Faculty of Medicine, Université de Montréal, Montréal, Quebec, Canada.

[5] LIS3D Laboratory, Sainte-Justine University Hospital Research Center, Montréal, Quebec, Canada.

[6] Orthopedic Division, Sainte-Justine University Hospital and Department of Surgery, Faculty of Medicine, Université de Montréal, Montréal, Quebec, Canada.

[7] Orthopedic Division, The Montreal Children's Hospital, Department of Surgery, McGill University, Montréal, Quebec, Canada.

[8] IRCCS Istituto Ortopedico Galeazzi, Milano, Italy.

[9] Department of Physiology and Pharmacology, Gdańsk University of Physical Education & Sport, Gdańsk, Poland.

[10] Vita-Salute San Raffaele University, Milano, Italy.

[11] Department of Biological Sciences, California State University, Chico, CA, USA

[12] Department of Stomatology, Faculty of Dentistry, Université de Montréal, Montréal, Quebec, Canada.

*Correspondence and requests for materials should be addressed to A.M. (email: alain.moreau@recherche-ste-justine.qc.ca)

Supplementary Figure and Table Legends

Supplementary Figure S1. Familial pedigrees showing the inheritance of AIS biological endophenotype. Family members affected with a scoliosis are in black.

Supplementary Figure S2. Variations in the effect of Pertussis toxin (PTX) on the response to various selective agonists of Gi-coupled receptors. (**A-J**) Osteoblasts from control subjects or AIS patients of the FG1, FG2, and FG3 groups were pre-incubated with (0.3 ng/mL, 1 ng/mL, 3 ng/mL, 10 ng/mL, 30 ng/mL, 100ng/mL, 1ug/mL) of PTX for 16 h prior to the stimulation with 10 µM of a specific synthetic agonist. The tested agonists and targeted receptors are indicated in each panel. The data are normalised to the response in the presence of ethanol and are expressed as mean ± SEM of the total number of values obtained from three independent experiments for n = 12 patients per group. * $P < 0.05$, **$P < 0.01$, ***$P < 0.001$, versus control group based on one-way ANOVA followed by a post-hoc Dunnett's test.

Supplementary Figure S3. Inhibition effect of GPAnt-2 on the response to various selective agonists of Gi-coupled receptors. (**A-J**) Osteoblasts from the control subjects or AIS patients of the different groups were pre-incubated with (10 nM, 100 nM, 1 µM, 10 µM, 1 mM, 10 mM) of GPAnt-2 for 1 h prior to the stimulation with 10 µM of a specific synthetic agonist. The tested agonists and targeted receptors are indicated in each panel. The data were normalised to the response obtained in the presence of the vehicle and are expressed as mean ± SEM of the total number of values obtained from three independent experiments for n = 12 patients per group.

Supplementary Figure S4. Stratifying the AIS functional groups based on the degree of response to various specific agonists of Gi-coupled receptors in myoblasts. (A-J) Agonists and targeted receptors are indicated in each panel. The data are normalised to the response of cells from control subjects in the presence of the vehicle (PBS) and are expressed as mean ± SEM of the total number of values obtained from three independent experiments for n = 12 patients per group.

Supplementary Figure S5. Stratifying the AIS functional groups based on the degree of the response to various specific agonists of the Gi-coupled receptors in PBMCs. (A-J) The agonists and targeted receptors are indicated in each panel. The data are normalised to the response of cells from control subjects in the presence of the vehicle (PBS) and are expressed as mean ± SEM of the total number of values obtained from three independent experiments for n = 12 patients per group.

Supplementary Figure S6. Validating the effectiveness of the Gαs and Gαq siRNA in AIS osteoblasts. (**A**) Total RNA extracted from the control and the AIS osteoblasts transfected with scramble, Gαs, or Gαq siRNA, and from non-transfected (NT) cells, were subjected to qRT-PCR analysis, using β-actin as the internal control. The data are expressed as mean ± SEM of the values obtained from n = 12 patients for each group. * $P < 0.05$, **$P < 0.01$, ***$P < 0.001$, versus NT cells based on one-way ANOVA followed by a post-hoc Dunnett's test. (**B**) The total cell lysates from cells transfected with scramble, Gs or Gq siRNA, were subjected to western blot analysis, using antibody to α-tubulin as control. The bands shown are representative of the results obtained with osteoblasts from 12 different patients for each AIS group. *The images are a representation of the original blots and full-length blots are presented in Supplementary Figure S13. Each strip originates from a distinct original gel, and is stacked for illustration purposes. Experiments were run in parallel and under the same experimental conditions*.

Supplementary Figure S7. Differential effects of siRNA knockdown of Gαs and Gαq on the biphasic impedance signature of the Gi-coupled receptors agonists among the AIS groups. The osteoblasts from the control subjects and the AIS patients of each functional group were transfected with scramble siRNA, Gαs siRNA, or Gαq siRNA, as indicated in the Materials and Methods section. The efficiency of siRNA in the control and the AIS groups was verified with qRT-PCR and Western blot analyses 48 hours after transfection. The response to cluster I, cluster II, cluster III, and cluster IV Gi-coupled receptor agonist stimulation was evaluated by challenging cells with 10 µM each of (**A, E, I, M**) LPA, (**B, F, J, N**) CB65, (**C, G, K, O**) Somatostatin, and (**D, H, L, P**) Apelin-17, respectively. The impedance represented in the y-axis as dziec was measured every 2 sec. The data are representative of the impedance signatures

generated by the CellKey™ system in the osteoblasts from each of the individuals in the control and AIS functional groups (n = 12 per group). Duplicate values are obtained from each of three independent experiments.

Supplementary Figure S8. AIS is not associated with changes in G protein expression or protein synthesis. (**A**) Total RNA were extracted from the osteoblasts of the control and the 3 AIS functional groups. The mRNA expression levels of the $G\alpha i_1$, $G\alpha i_2$, $G\alpha i_3$, Gαs, and Gαq genes in the control relative to the AIS functional groups, were compared with qRT-PCR. β-actin was used as an internal control. The error bars show SEM for n = 12 patients per group. (**B**) The lysates were obtained from the osteoblasts of the control subjects and the AIS patients. Equal amounts of proteins (40 µg) of each lysate were resolved by 10% SDS-PAGE and immunoblotted for specific antibodies against the indicated proteins. The bands shown are representative of the results obtained with the osteoblasts from 12 different patients for each group. *The images are a representation of the original blots and full-length blots are presented in Supplementary Figure S14. Each strip originates from a distinct original gel and is stacked for illustration purposes. Experiments were run in parallel and under the same experimental conditions*.

Supplementary Figure S9. Validation of the effectiveness and the selectivity of siRNA of each Gαi isoform (**A**) The total RNA extracted from the control and AIS osteoblasts transfected with scramble, $G\alpha i_1$, $G\alpha i_2$, or $G\alpha i_3$ siRNA, and non-transfected (NT) cells, were subjected to qRT-PCR analysis, using β-actin as an internal control. The data are expressed as mean ± SEM of the values obtained from n = 12 patients for each group. * $P < 0.05$, ** $P < 0.01$, *** $P < 0.001$,

versus NT cells based on one-way ANOVA followed by a post-hoc Dunnett's test. (**B**) The total cell lysates from cells transfected with scramble, Gi_1, Gi_2, or Gi_3 siRNA, were subjected to western blot analysis, using an antibody to α-tubulin as a control. The bands shown are representative of the results obtained with the osteoblasts from 12 different patients for each AIS group. *The images are a representation of the original blots and full-length blots are presented in Supplementary Figure S15. Each strip originates from a distinct original gel and is stacked for illustration purposes. Experiments were run in parallel and under the same experimental conditions*.

Supplementary Figure S10. Differential effects of Gαi and Gαs siRNA knockdown on the response to Gi stimulation in the AIS functional groups. Osteoblasts from the (**A**) control subjects, (**B**) FG1, (**C**) FG2, and (**D**) FG3 were transfected with $G\alpha i_1$, $G\alpha i_2$, $G\alpha i_3$, Gαs, Gαq siRNA alone or in combination, or with scramble siRNA, as indicated in the Materials and Methods section. The efficiency of the siRNAs was verified with qRT-PCR and western blot analyses in each functional group 48 hours after transfection, and the response to Gi stimulation was evaluated by challenging the cells with 10 µM of melatonin or LPA. The data are normalised to the response in the cells transfected with scramble siRNA, and are expressed as mean ± SEM of the values obtained from n = 12 patients for each group. * $P < 0.05$, **$P < 0.01$, ***$P < 0.001$, versus control group based on one-way ANOVA followed by a post-hoc Dunnett's test.

Supplementary Figure S11. Original blots and full-length blots used to generate the Fig. 6. *Experiments were run in parallel and under the same experimental conditions*.

Supplementary Figure S12. Original blots and full-length blots used to generate the Fig. 7. *Experiments were run in parallel and under the same experimental conditions.*

Supplementary Figure S13. Original blots and full-length blots used to generate the Supplementary Figure S6. *Experiments were run in parallel and under the same experimental conditions*.

Supplementary Figure S14. Original blots and full-length blots used to generate the Supplementary Figure S8. *Experiments were run in parallel and under the same experimental conditions*.

Supplementary Figure S15. Original blots and full-length blots used to generate the Supplementary Figure S9. *Experiments were run in parallel and under the same experimental conditions*.

Supplementary Table S1. Effect of various Gi-coupled receptor agonists on the impedance response in the osteoblasts from control and AIS patients. EC_{50} values were calculated from the concentration-response curves by non-linear regression analysis using the GraphPad prism. The values are expressed as mean ± SEM of the total number of values obtained from three independent experiments for n = 12 patients per group. ***P < 0.001, versus control group based on one-way ANOVA followed by a post-hoc Dunnett's test.

Supplementary Table S2. Potency of GPAnt2 to inhibit various Gi-coupled receptor agonists in the osteoblasts from control and AIS patients. IC_{50} values were calculated from the concentration-response curves by non-linear regression analysis using GraphPad Prism. The values are expressed as mean ± SEM of the total number of values obtained from three independent experiments for n = 12 patients per group. ***P < 0.001, versus control group based on one-way ANOVA followed by a post-hoc Dunnett's test.

Supplementary Table S3. List of siRNA oligonucleotides used for knockdown experiments

Supplementary Table S4. List of primers used for qRT-PCR expression analyses

Supplementary Table S5. Relative levels of serine phosphorylated Gαi protein isoforms in each AIS biological endophenotype

Supplementary Figure S1

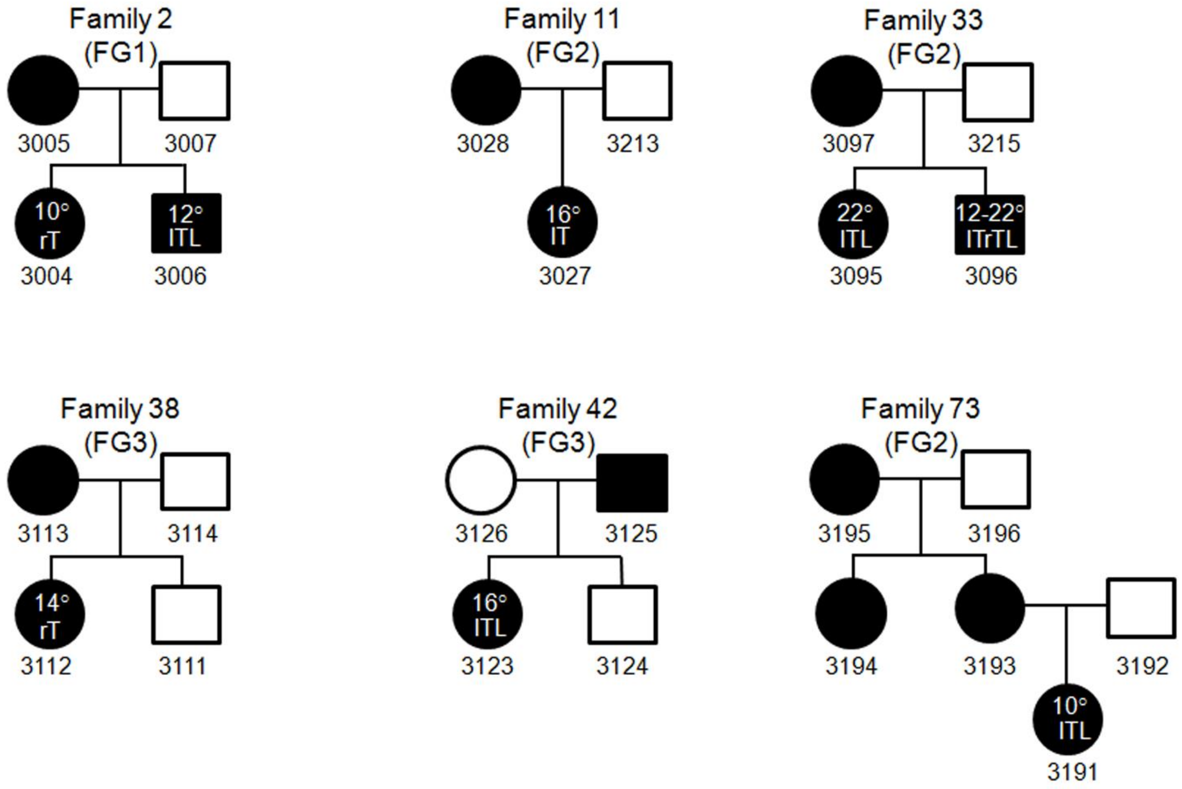

Supplementary Figure S2

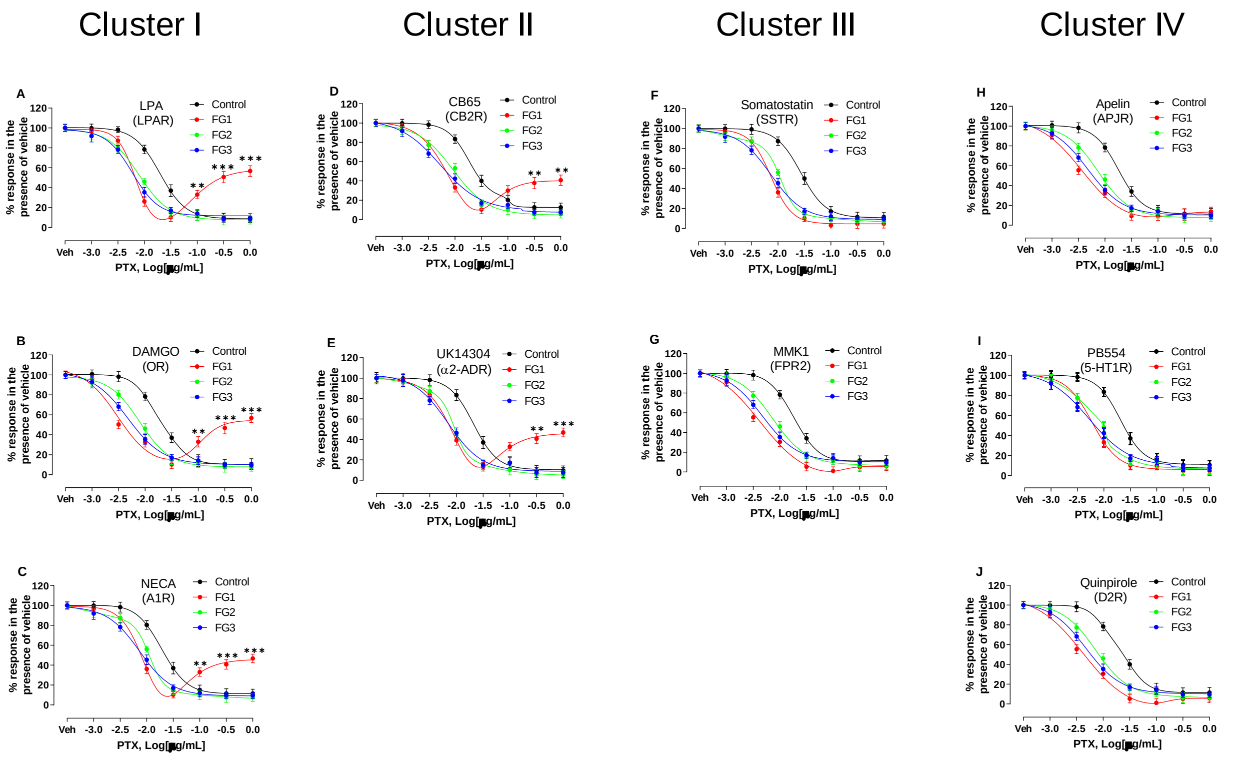

Supplementary Figure S3

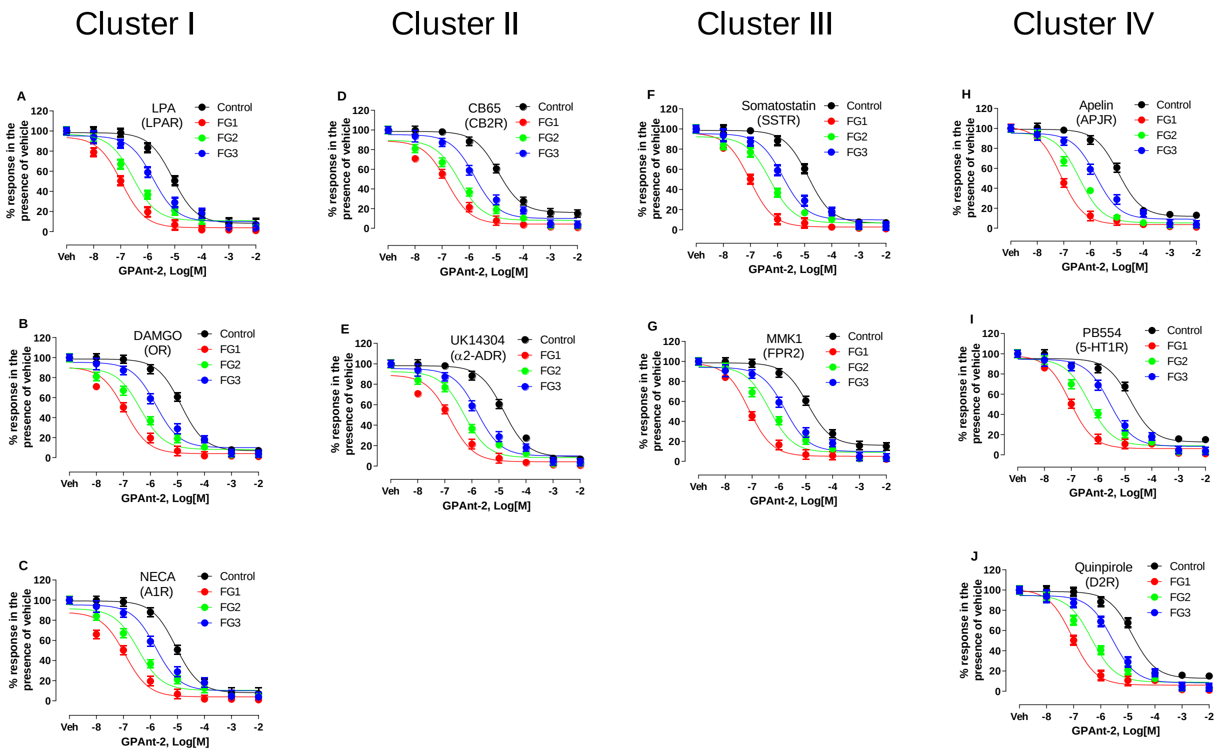

Supplementary Figure S4

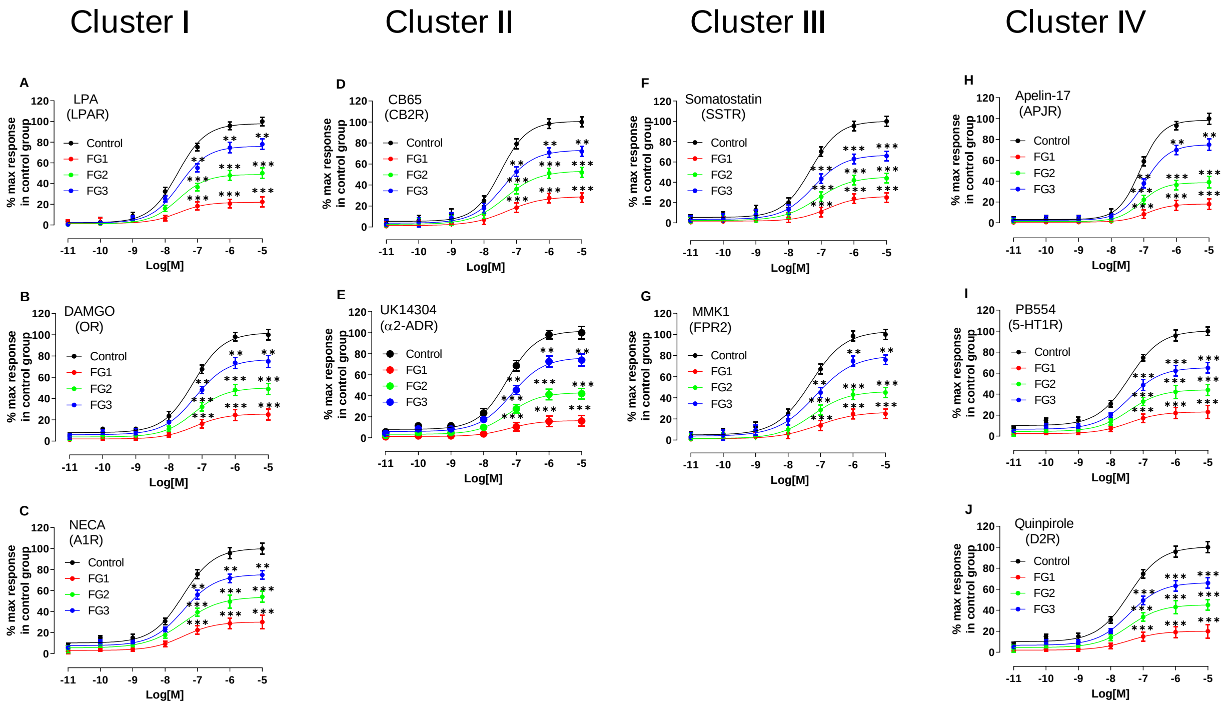

Supplementary Figure S5

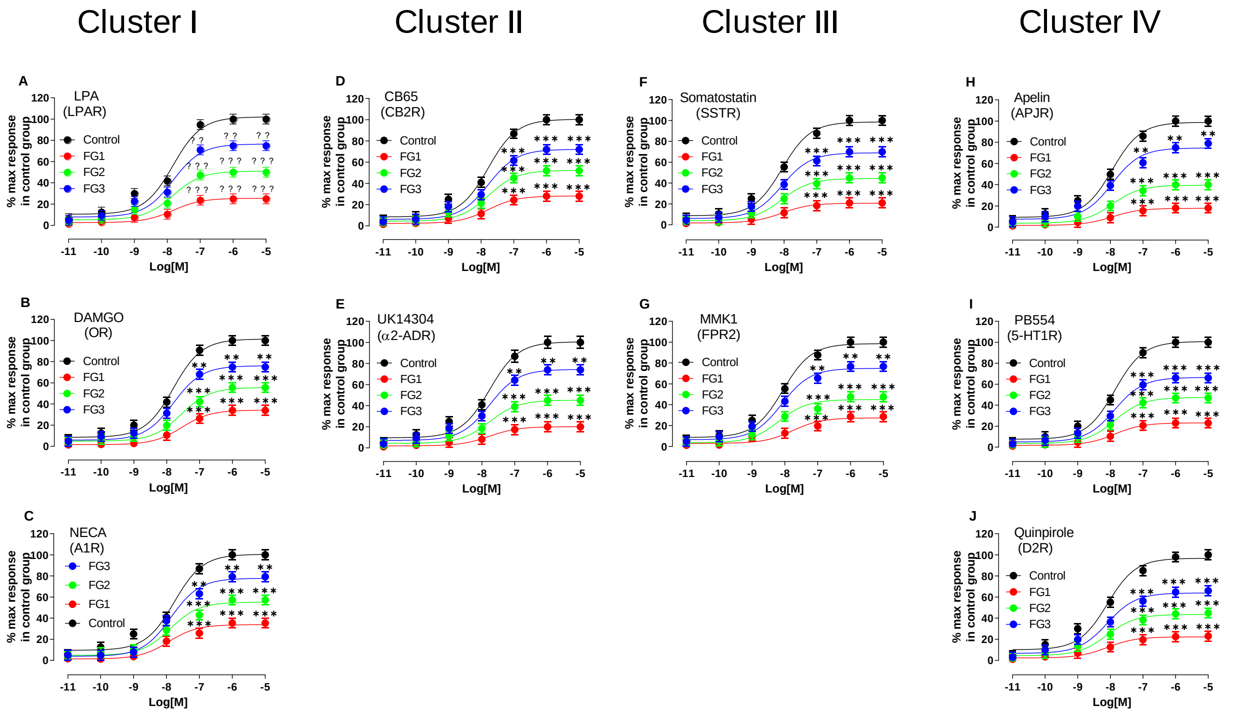

Supplementary Figure S6

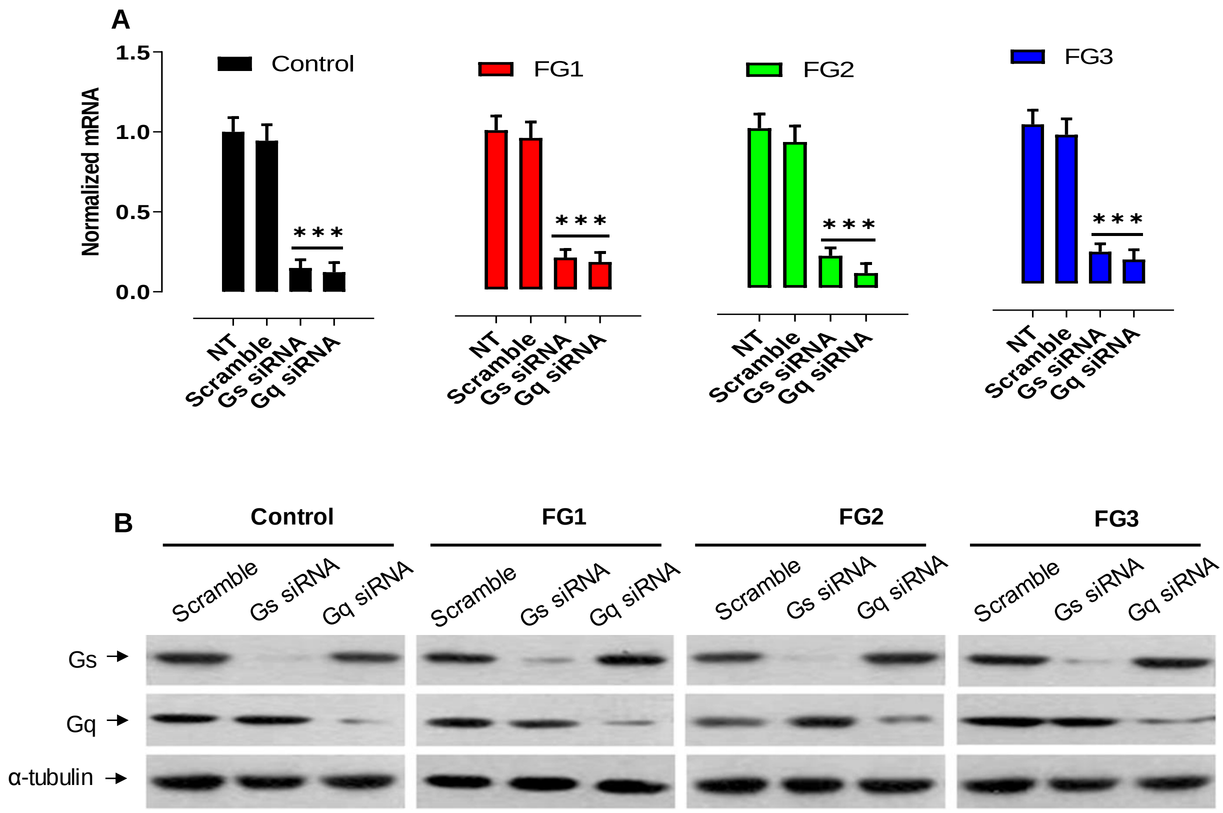

Supplementary Figure S7

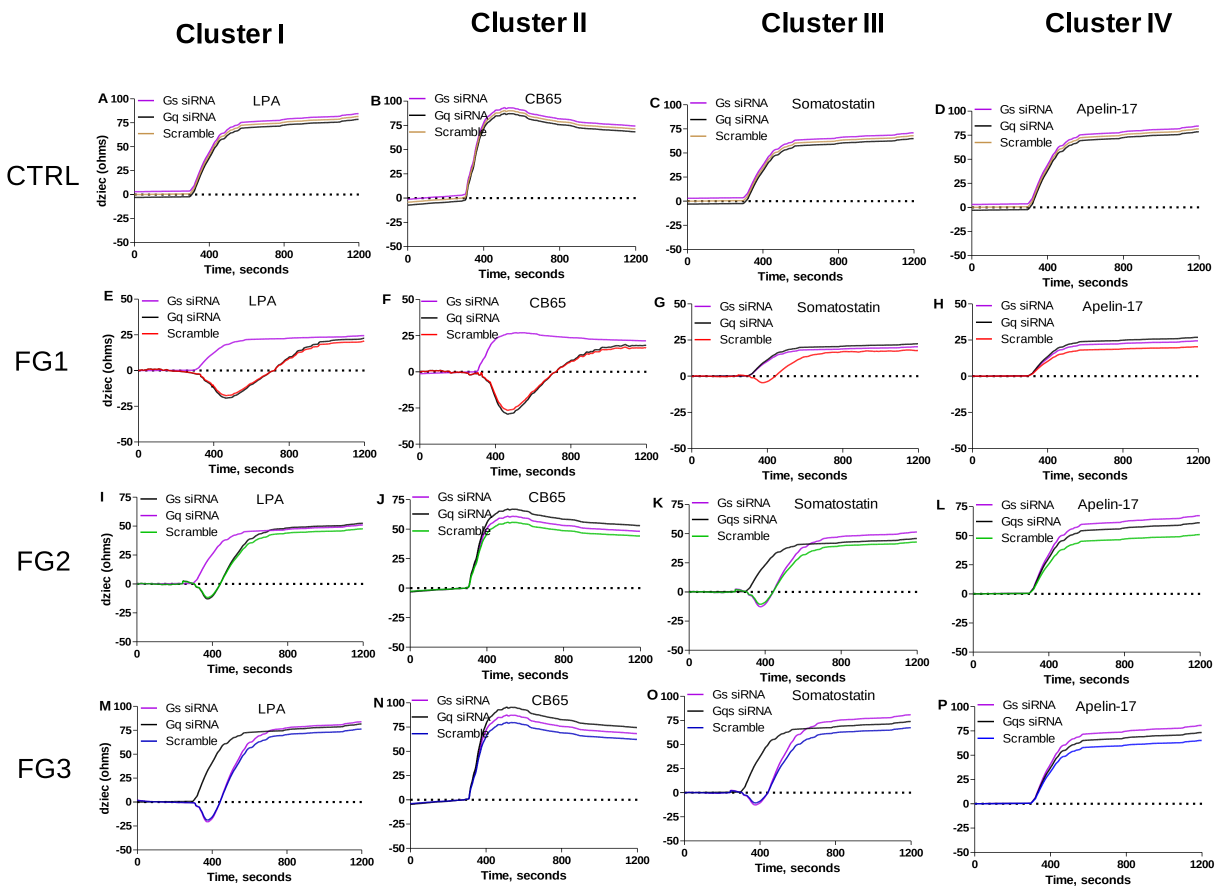

Supplementary Figure S8

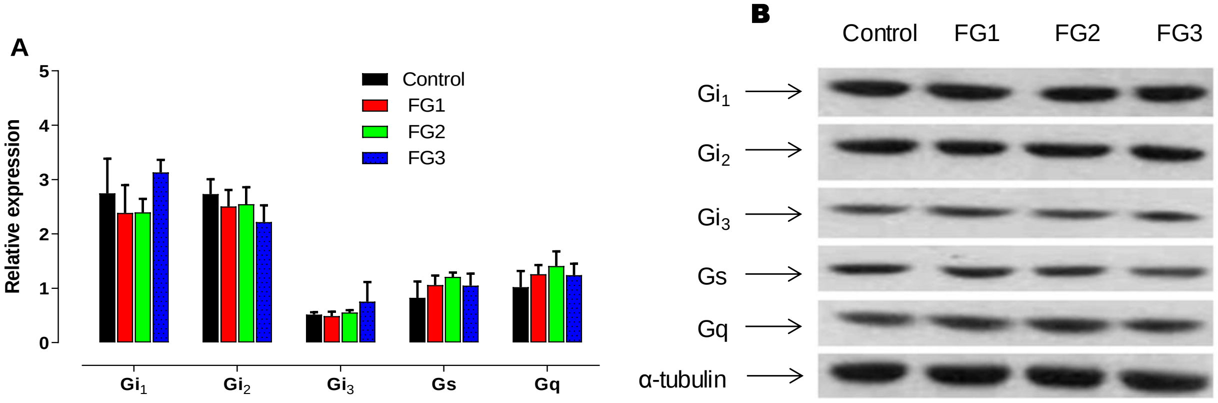

Supplementary Figure S9

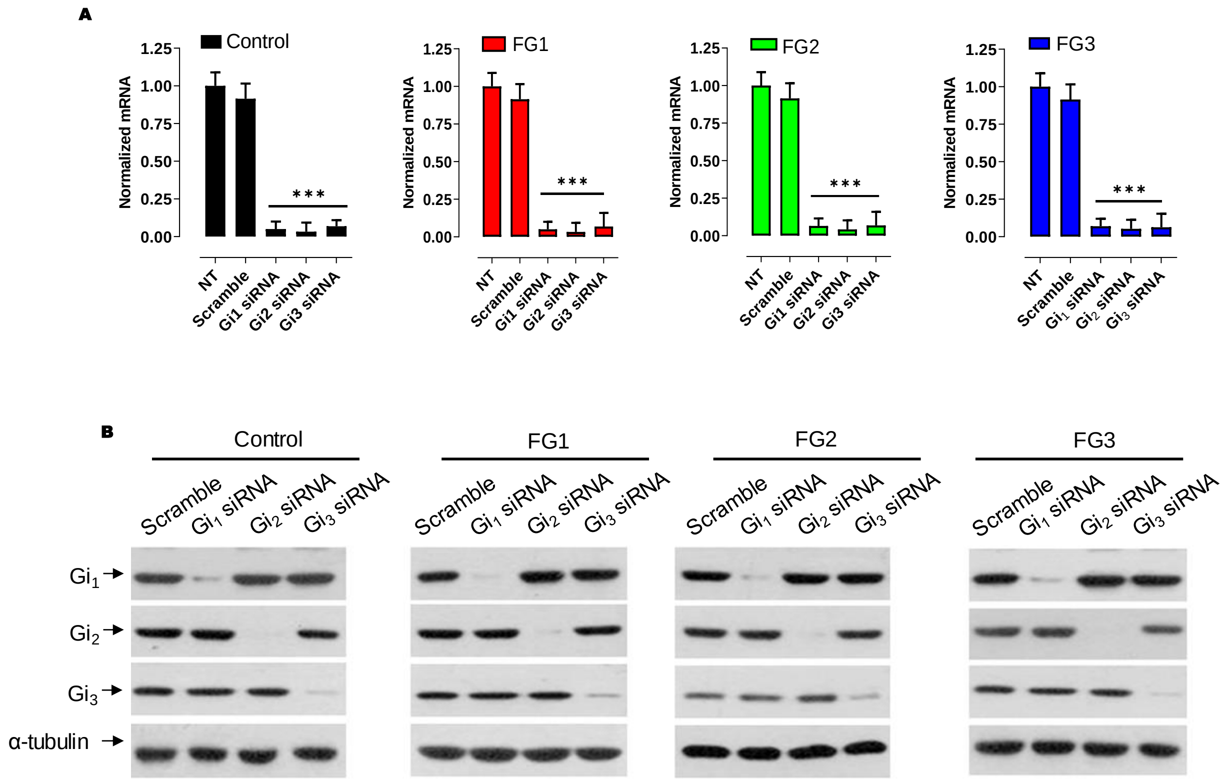

Supplementary Figure S10

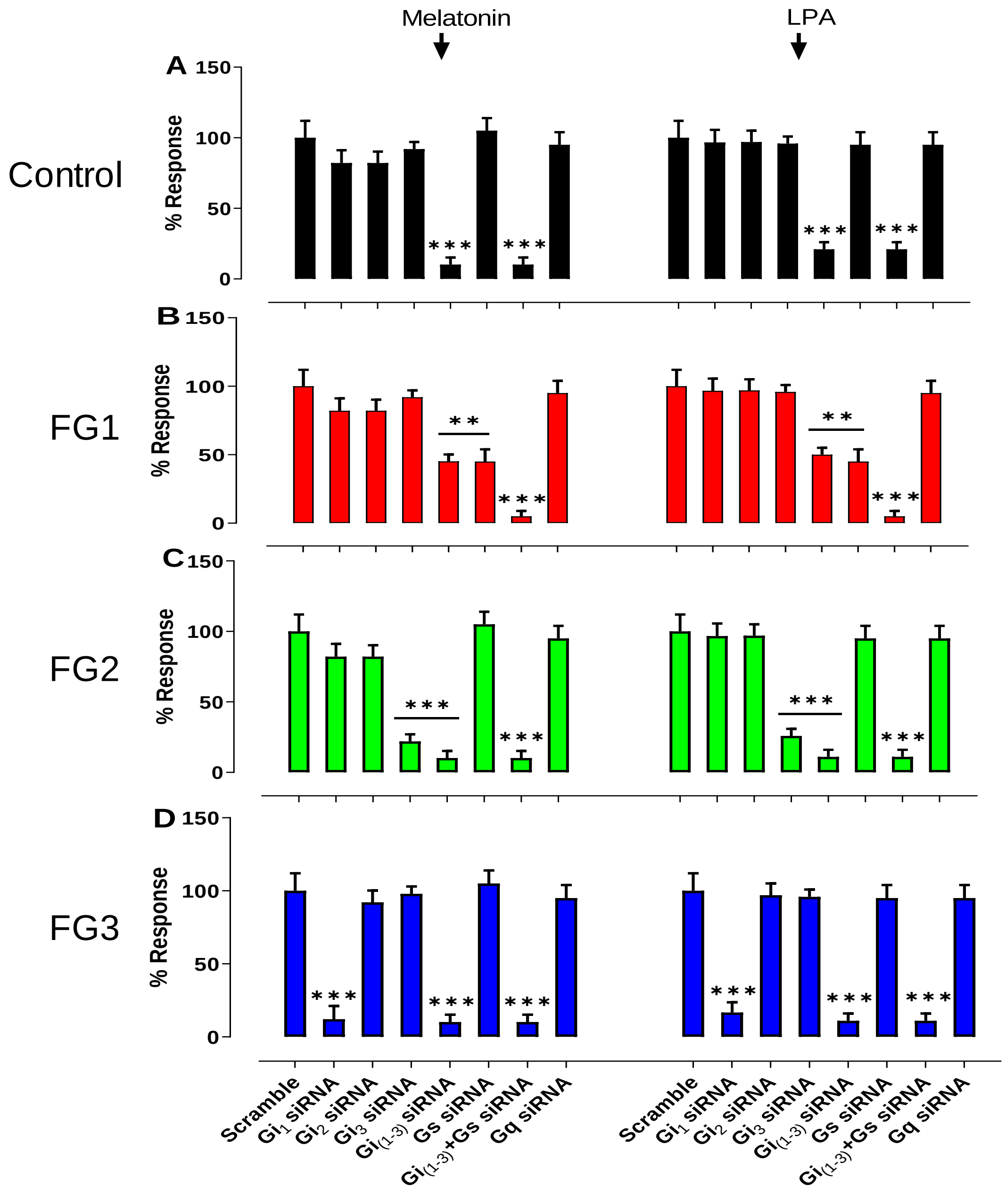

Supplementary Figure S11

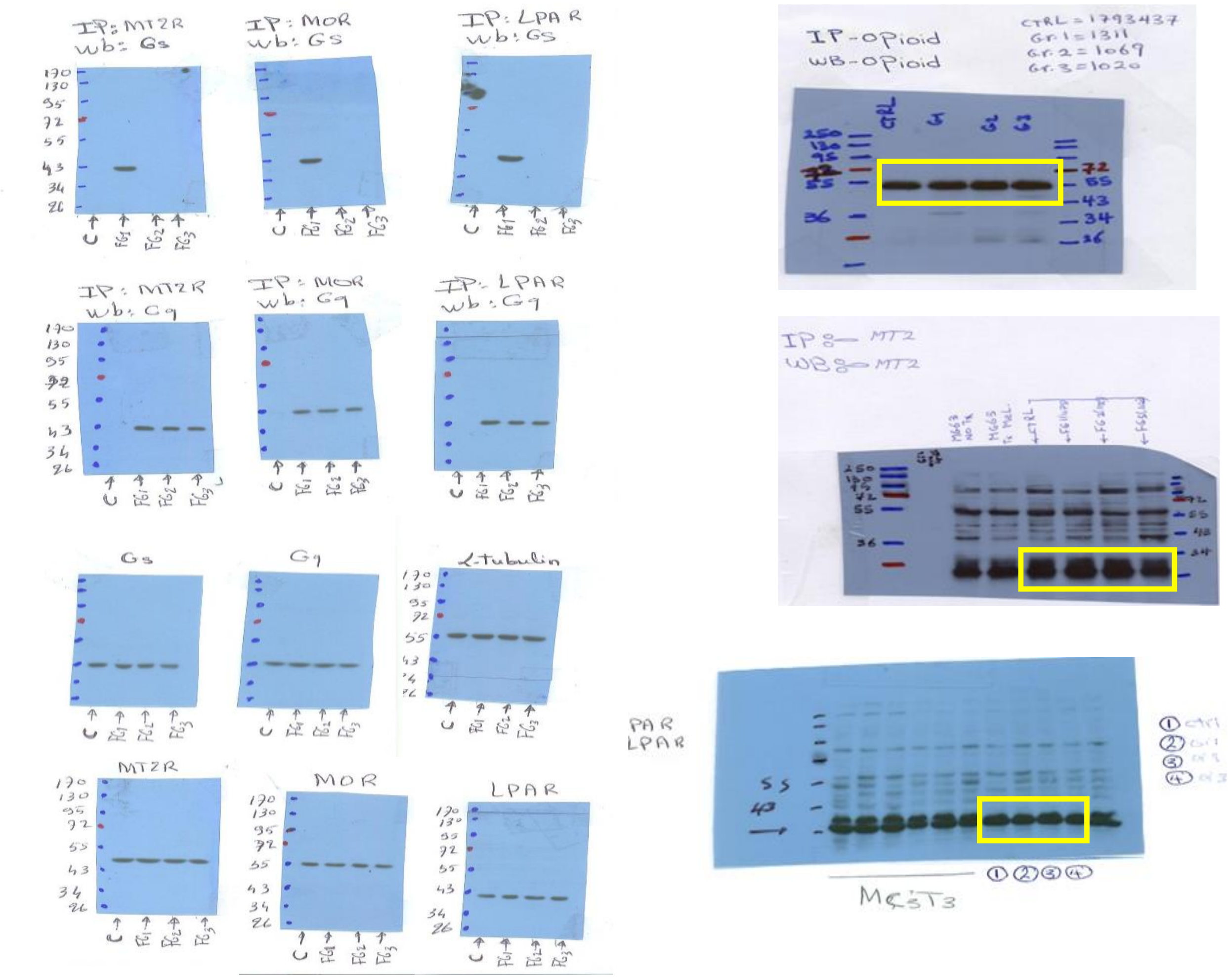

Supplementary Figure S12

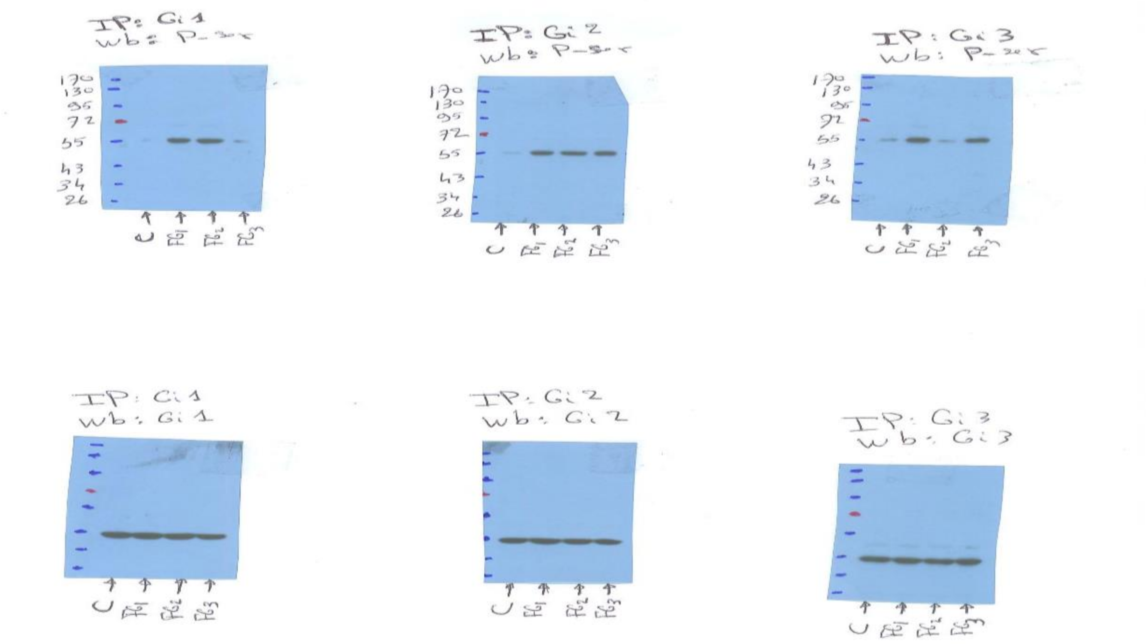

Supplementary Figure S13

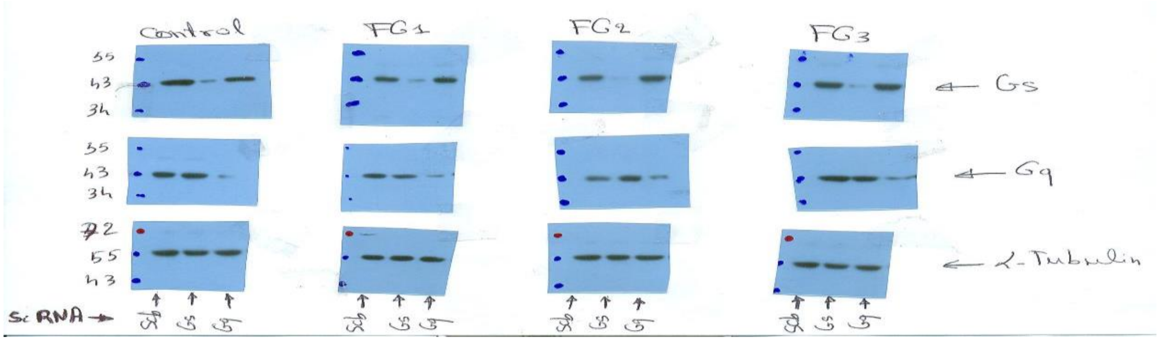

Supplementary Figure S14

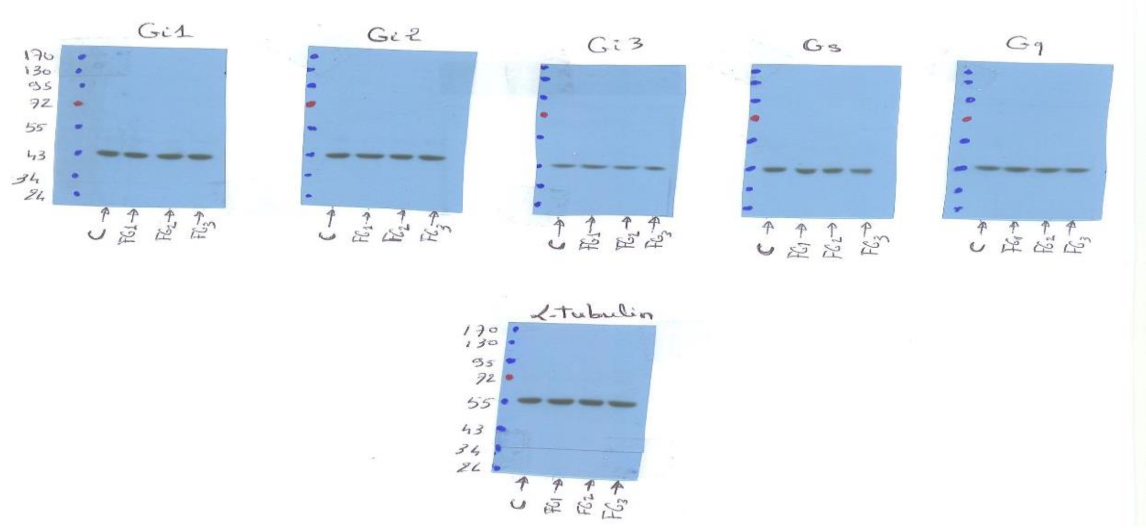

Supplementary Figure S15

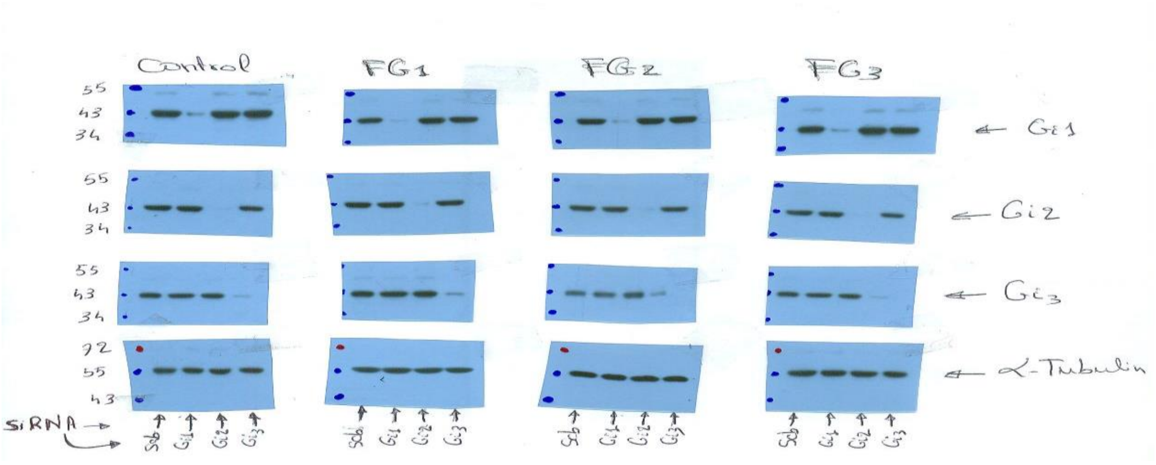

Supplementary Table S1. Effect of various Gi-coupled receptor agonists on the impedance response in the osteoblasts from control and AIS patients

	Control	FG1	FG2	FG3
	EC_{50} (nM)	EC_{50} (nM)	EC_{50} (nM)	EC_{50} (nM)
**Melatonin**	33.4 ± 8.4	42.3 ± 9.4	48.68 ± 6.4	45.79 ± 5.3
**LPA**	8.52 ± 1.5	8.78 ± 0.6	8.54 ± 1.4	8.59 ± 1.2
**DAMGO**	18.78 ± 2.2	18.67 ± 2.1	19.11 ± 2.3	19.88 ± 2.4
**NECA**	20.85 ± 2.2	20.88 ± 1.7	20.83 ± 1.2	22.25 ± 2.2
**CB65**	13.91 ± 1.2	13.97 ± 1.2	13.7 ± 1.2	13.95 ± 1.3
**UK14304**	16.48 ± 2.3	18.52 ± 4.2	16.65 ± 1.6	17.8 ± 1.3
**Somatostatin**	31.12 ± 3.3	31.23 ± 3.1	31.3 ± 2.6	30.98 ± 2.3
**MMK1**	48.95 ± 4.3	50.51 ± 2.3	50.45 ± 5.2	51.58 ± 2.3
**Apelin-17**	32.50 ± 2.3	32.45 ± 2.6	32.67 ± 1.9	39.1 ± 2.4
**BP554**	22.30 ± 1.7	22.33 ± 2.3	22.50 ± 1.6	22.37 ± 1.5
**Quinpirole**	43.57 ± 4.1	45.39 ± 4.2	47.1 ± 3.2	48.7± 5.1

EC_{50} values were calculated from the concentration-response curves by non-linear regression analysis using the GraphPad prism. The values are expressed as mean ± SEM of the total number of values obtained from three independent experiments for n = 12 patients per group. ***P < 0.001, versus control group based on one-way ANOVA followed by a post-hoc Dunnett's test.

Supplementary Table S2. Potency of GPAnt2 to inhibit various Gi-coupled receptor agonists in the osteoblasts from control and AIS patients

	Control	FG1	FG2	FG3
	IC_{50} (uM)	IC_{50} (uM)	IC_{50} (uM)	IC_{50} (uM)
**Melatonin**	11.76 ± 0.2	0.06 ± 0.01***	0.26 ± 0.03***	1.17 ± 0.4***
**LPA**	7.88 ± 2.2	0.11 ± 0.03***	0.38 ± 0.2***	1.33 ± 0.2***
**DAMGO**	13.40 ± 0.1	0.1 ± 0.01***	0.32± 0.1***	1.58± 0.2***
**NECA**	6.72 ± 0.1	0.11 ± 0.01***	0.42 ± 0.1***	1.45 ± 0.1***
**CB65**	11.26 ± 0.3	0.14 ± 0.05***	0.39 ± 0.1***	1.38 ± 0.2***
**UK14304**	12.35 ± 0.2	0.15 ± 0.02***	0.64 ± 0.3***	1.76 ± 0.3***
**Somatostatin**	13.5 ± 0.2	0.09 ± 0.02***	0.52 ± 0.3***	1.54 ± 0.3***
**MMK1**	6.22 ± 0.2	0.08 ± 0.02***	0.44 ± 0.2***	1.88 ± 0.3***
**Apelin-17**	10.33 ± 0.3	0.08 ± 0.01***	0.30 ± 0.03***	1.49 ± 0.3***
**BP554**	15.07 ± 0.2	0.1 ± 0.02***	0.45 ± 0.3***	2.67 ± 0.3***
**Quinpirole**	8.82 ± 0.1	0.1 ± 0.03***	0.33 ± 0.2***	1.78 ± 0.2***

IC_{50} values were calculated from the concentration-response curves by non-linear regression analysis using GraphPad Prism. The values are expressed as mean ± SEM of the total number of values obtained from three independent experiments for n = 12 patients per group. ***P < 0.001, versus control group based on one-way ANOVA followed by a post-hoc Dunnett's test.

298

Supplementary Table S3. List of siRNA oligonucleotides used for knockdown experiments

Gene symbol	siRNA sequence	Cat. Number
GNAi1	GAGAUUGUGGAAAGAUAGUGGUGUA	1299003
GNAi2	GAGGACCUGAAUAAGCGCAAAGACA	1299003
GNAi3	UCAGCUCAAUGAUUCUGCUUCAUAU	1299003
GNAS	ACAACAUGGUCAUCCGGGAGGACAA	1299003
GNAQ	GGAGAGAGUGGCAAGAGUACGUUUA	10620319

Supplementary Table S4. List of primers used for qRT-PCR expression analyses

	Forwards	reverses
Gi1	AGGGCTATGGGGAGGTTGAAGAT	ACTCCAGCAAGTTCTGCAGTCA
Gi2	AGGGAATACCAGCTCAACGACTCA	TGTGTGGGGATGTAGTCACTCTGT
Gi3	GAGAGTGAAGACCACAGGCATT	CGTTCTGATCTTTGGCCACCTA
Gs	GAGACCAAGTTCCAGGTGGACA	GATCCACTTGCGGCGTTCAT
Gq	ATCAGAACATCTTCACGGCC	AAAGCAGACACCTTCTCCAC

Supplementary Table S5. Relative levels of serine-phosphorylated Gαi protein isoforms in each AIS biological endophenotype

	Control	FG1	FG2	FG3
P-ser-th/Gi1	0.077 ± 0.008	1.192 ± 0.052***	1.154 ± 0.054***	0.113 ± 0.042
P-ser-th/Gi2	0.059 ± 0.007	1.141 1 ± 0.036***	1.088 ± 0.022***	1.213 ± 0.044***
P-ser-th/Gi3	0.032 ± 0.002	1.153 ± 0.047***	0.0456 ± 0.005	1.147 ± 0.051***
